# Copper-catalyzed trifluoromethylation of alkenes with an electrophilic trifluoromethylating reagent

**DOI:** 10.3762/bjoc.9.299

**Published:** 2013-11-25

**Authors:** Xiao-Ping Wang, Jin-Hong Lin, Cheng-Pan Zhang, Ji-Chang Xiao, Xing Zheng

**Affiliations:** 1 Key Laboratory of Organofluorine Chemistry, Shanghai Institute of Organic Chemistry, Chinese Academy of Sciences,345 Lingling Road, Shanghai 200032, China; 2 Institute of Pharmacy and Pharmacology, University of South China, Hengyang, Hunan 421001, China

**Keywords:** alkenes, catalysis, copper, electrophilic trifluoromethylating reagent, trifluoromethylation

## Abstract

An efficient method for the copper-catalyzed trifluoromethylation of terminal alkenes with an electrophilic trifluoromethylating reagent has been developed. The reactions proceeded smoothly to give trifluoromethylated alkenes in good to excellent yields. The results provided a versatile approach for the construction of C_vinyl_–CF_3_ bonds without using prefunctionalized substrates.

## Introduction

**Scheme 1:**
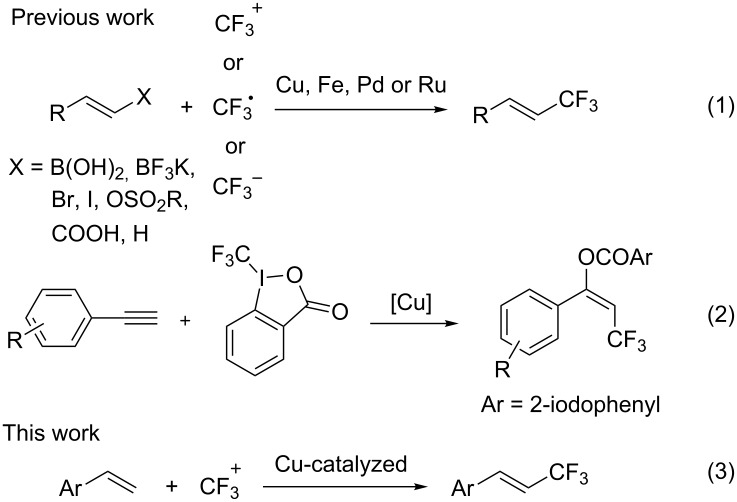
Construction of the C_vinyl_–CF_3_ bond.

**Scheme 2:**
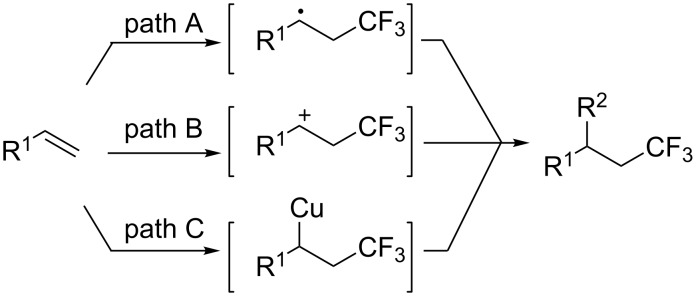
Proposed reaction paths for the trifluoromethylation of alkenes.

The incorporation of a trifluoromethyl group into pharmaceutically and agrochemically relevant molecules usually enhances their chemical and metabolic stability, lipophilicity and binding selectivity [1–7]. As a result, considerable effort has been directed towards the development of efficient and versatile trifluoromethylation methods [8–16]. The past few years has witnessed the rapid advances in transition metal-promoted trifluoromethylation for the construction of C_aryl_–CF_3_ bonds [17–31]. In contrast, transition metal-mediated trifluoromethylation to form C_vinyl_–CF_3_ bonds has been much less explored. As illustrated in Scheme 1, the strategies developed recently usually require the use of prefunctionalized alkenes, which could be classified into the following: vinylboronic acids, vinyl borates, vinyl halides, vinyl sulfonates and vinyl carboxylic acids (Scheme 1, reaction 1) [32–38]. Cho and co-workers reported a radical process for the trifluoromethylation of terminal alkenes without using prefunctionalized substrates, but a volatile reagent was used (Scheme 1, reaction 1) [39]. Szabó described trifluoromethyl-benzoyloxylation of alkynes to construct C_vinyl_–CF_3_ bonds (Scheme 2, reaction 2) [40]. As part of our continuing interest in trifluoromethylation reactions [25,41–45], we investigated the copper-catalyzed trifluoromethylation of terminal alkenes with electrophilic trifluoromethylating reagents (Scheme 1, reaction 3).

Transition metal-catalyzed trifluoromethylation of terminal alkenes has been reported by several groups. However, these methods focused on the formation of C_sp3_–CF_3_ bonds [46–56]. In copper-catalyzed reactions with electrophilic trifluoromethylating reagents, it was proposed that the transformation might proceed via a radical, electrophilic or Heck-type process (Scheme 2) [47–50]. We reasoned that even if the reaction involved the radical process (path A, Scheme 2), the radical intermediate could still be oxidized to a cation because electrophilic trifluoromethylation reagents can be considered as an oxidant. In the presence of base, both of the cation and Heck-type intermediates should be able to undergo hydrogen elimination to form a C_vinyl_–CF_3_ bond. On the basis of these reports and our hypothesis, we commenced to examine the reaction of aromatic alkenes with electrophilic trifluoromethylation reagents in the presence of copper and base.

## Results and Discussion

Previously, we reported that copper powder or cuprous iodide could promote trifluoromethylation of heteroaromatics, arylboronic acids or terminal alkynes with trifluoromethyl sulfonium salt **I** [25,44–45,57–58]. But the same trifluoromethylation reagent (2 equiv) failed to convert 4-vinylbiphenyl to the expected alkene in reasonable yields in acetonitrile with B_1_ (DBU) as the base, even though cuprous iodide gave better results (Table 1, entries 1 and 2). In the presence of cuprous iodide, Umemoto’s reagent (**II**) and Togni’s reagent (**III**) were found to be more efficient in this transformation (Table 1, entries 3 and 4). ^19^F NMR measurements showed that the reaction system turned to be complex and low yield of the desired product was determined when another Togni’s reagent (**IV**) was used (Table 1, entry 5). Other cuprous complexes were also studied with the use of Togni’s reagent (**III**) (Table 1, entries 6–10). Better result obtained with [(MeCN)_4_Cu]PF_6_ prompted us to continue to use this copper source (Table 1, entry 10). When B_2_ was used instead of B_1_, almost no desired product was observed (Table 1, entry 11). That might be because B_2_ not only acted as base, but also acted as strongly coordinating ligand, which poisoned the catalyst and shut down the reaction. Other bases (Table 1, entries 12–15), including an inorganic base (Table 1, entry 15), failed to accelerate the desired conversion either. The examination of the solvent effect showed that the solvent is quite important for the reaction (Table 1, entries 16–20). When the reaction was carried out in DMF, the expected product **2a** was obtained in excellent yield (Table 1, entry 16). The yield was decreased dramatically in DMSO (Table 1, entry 17). Almost no reaction took place in polar protic solvent (Table 1, entry 18) and moderate results were achieved in less polar solvents (Table 1, entries 19 and 20). With increasing the amount of Togni’s reagent (**III**) in the suitable solvent, DMF, the product was obtained almost in quantitative yield with excellent stereoselectivity (dr >98:2) determined by ^19^F NMR (Table 1, entry 21). Decreasing the amount of this reagent resulted in a lower yield (Table 1, entry 22). The absence of catalyst or base led to no reaction or incredibly low yield, which means both of them are crucial for the reaction (Table 1, entries 23–25).

**Table 1:** Trifluoromethylation of 4-vinylbiphenyl by electrophilic trifluoromethylation reagents.

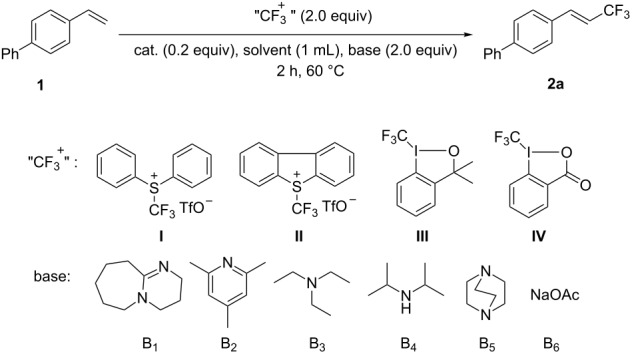					

Entry	Cat.	Base	Solvent	“CF_3_^+^”	Yield (%)^a^

1	Cu	B_1_	CH_3_CN	**I**	trace
2	CuI	B_1_	CH_3_CN	**I**	30
3	CuI	B_1_	CH_3_CN	**II**	56
4	CuI	B_1_	CH_3_CN	**III**	60
5	CuI	B_1_	CH_3_CN	**IV**	30
6	CuBr	B_1_	CH_3_CN	**III**	46
7	CuCl	B_1_	CH_3_CN	**III**	36
8	CuTc	B_1_	CH_3_CN	**III**	54
9	CuOAc	B_1_	CH_3_CN	**III**	41
10	[(MeCN)_4_Cu]PF_6_	B_1_	CH_3_CN	**III**	75
11	[(MeCN)_4_Cu]PF_6_	B_2_	CH_3_CN	**III**	trace
12	[(MeCN)_4_Cu]PF_6_	B_3_	CH_3_CN	**III**	10
13	[(MeCN)_4_Cu]PF_6_	B_4_	CH_3_CN	**III**	trace
14	[(MeCN)_4_Cu]PF_6_	B_5_	CH_3_CN	**III**	10
15	[(MeCN)_4_Cu]PF_6_	B_6_	CH_3_CN	**III**	7
16	[(MeCN)_4_Cu]PF_6_	B_1_	DMF	**III**	90
17	[(MeCN)_4_Cu]PF_6_	B_1_	DMSO	**III**	57
18	[(MeCN)_4_Cu]PF_6_	B_1_	CH_3_OH	**III**	trace
19	[(MeCN)_4_Cu]PF_6_	B_1_	THF	**III**	67
20	[(MeCN)_4_Cu]PF_6_	B_1_	CHCl_3_	**III**	75
21^b^	[(MeCN)_4_Cu]PF_6_	B_1_	DMF	**III**	98
22^c^	[(MeCN)_4_Cu]PF_6_	B_1_	DMF	**III**	58
23	–	–	DMF	**III**	0
24	–	B_1_	DMF	**III**	0
25	[(MeCN)_4_Cu]PF_6_	–	DMF	**III**	10

^a^Yields determined by ^19^ F NMR spectroscopy. ^b^2.5 equiv of **III** was used. ^c^1.5 equiv of **III** was used.

**Figure 1:**
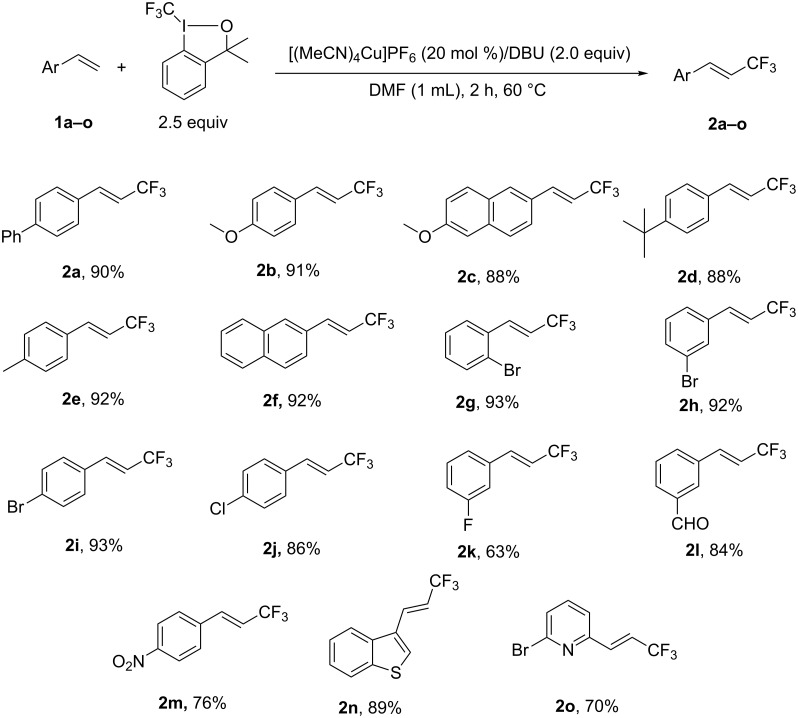
Cu(I)-catalyzed trifluoromethylation of terminal alkenes with Togni’s reagent. Isolated yield are recorded.

With the optimized reaction conditions in hand (Table 1, entry 21), we then explored the substrate scope of the Cu(I)-catalyzed trifluoromethylation of terminal alkenes with Togni’s reagent. As shown in Figure 1, the reaction could tolerate various functional groups. It is worth mentioning that all of the products were obtained with excellent stereoselectivity (*E*/*Z* >97/3), determined by ^19^F NMR. Substrates with an electron-donating group were converted smoothly into the desired products in excellent yields (**2a**–**2f**). Irrespective of the position of the bromine substituent on the aryl ring, the reaction proceeded very well to afford the desired products in excellent yields (**2g**–**2i**). Stronger electron-withdrawing groups showed some negative effect on the reaction, as exemplified by the poor results of substrates with other halides, carbonyl or nitro groups on the benzene ring (**2j**–**2m**). Heteroaromatic alkenes were also investigated (**2n** and **2o**). As previously seen, alkene **1n** endowed with an electron-rich heteroaromatic group led to a good result (**2n**) and an electron-deficient substrate resulted in low yield (**2o**).

**Scheme 3:**
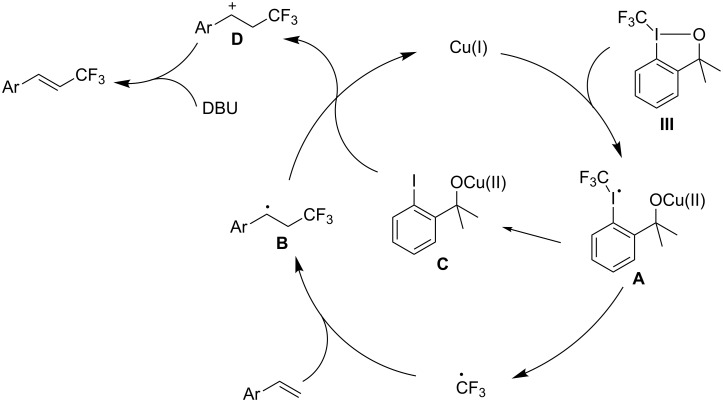
Proposed mechanism for the trifluoromethylation of terminal alkenes.

With regard to the reaction mechanism, it is reasonable to conceive a pathway involving radical species according to previous reports [47–50]. To gain more insight into the mechanism, further evidence was collected. 2,2,6,6-Tetramethyl-1-piperidinyloxy (TEMPO), a well-known radical scavenger, was added to the reaction of 4-vinylbiphenyl with Togni’s reagent (**III**) in the presence of [(MeCN)_4_Cu]PF_6_. It was found that the desired trifluoromethylation was completely suppressed, which suggested that the transformation involved a radical process. Based on the above results, we proposed the mechanism as outlined in Scheme 3. Initially, the activation of **III** by Cu(I) led to the formation of radical intermediate **A**. Decomposition of this intermediate produces ((2-(2-iodophenyl)propan-2-yl)oxy)copper(II) (**C**) and a CF_3_ radical, which is trapped by alkenes to form the trifluoromethylated radical intermediate **B**. Subsequently, the radical intermediate **B** is oxidized by Cu(II) (**C**) to the cation intermediate **D** with simultaneous release of catalyst Cu(I). In the presence of base, intermediate **D** readily undergoes hydrogen elimination to give the final product.

## Conclusion

In conclusion, we have described the copper-catalyzed trifluoromethylation of alkenes with Togni’s reagent under mild conditions. The results presented here provided a versatile approach for the construction of C_vinyl_–CF_3_ bonds without using prefunctionalized substrates. Investigations on the application of the trifluoromethylation method to the synthesis of pharmaceuticals and agrochemicals are currently underway.

## Supporting Information

File 1 Full experimental details, analytical data and spectra of the target compounds.
