# Supplementary material for: Copper-catalyzed trifluoromethylation of alkenes with an electrophilic trifluoromethylating reagent
Source: Beilstein J Org Chem. 2013 Nov 25;9:2635–40. doi: 10.3762/bjoc.9.299 (PMC3869243; doi:10.3762/bjoc.9.299)
Supplement: File 1 — Full experimental details, analytical data and spectra of the target compounds. [file Beilstein_J_Org_Chem-09-2635-s001.pdf]

Supporting Information

for

Copper-catalyzed trifluoromethylation of alkenes with an electrophilic trifluoromethylating reagent

Xiao-Ping Wang[1,2], Jin-Hong Lin[1], Cheng-Pan Zhang[1], Ji-Chang Xiao*[1] and Xing Zheng[2]

Address: [1]Key Laboratory of Organofluorine Chemistry, Shanghai Institute of Organic Chemistry, Chinese Academy of Sciences,345 Lingling Road, Shanghai 200032, China and [2]Institute of Pharmacy and Pharmacology, University of South China, Hengyang, Hunan 421001, China

Email: Ji-Chang Xiao* - jchxiao@sioc.ac.cn

* Corresponding author

Full experimental details, analytical data and spectra of the target compounds

Tables of Contents

1. General information……………………………………………………….. S2

2. General procedure for the trifluoromethylation of terminal alkenes………...…..S2

3. Copies of ^{1}H NMR, ^{19}F NMR, and ^{13}C NMR spectra of the target compounds **2a**–**2o** S6

1. General information

^{1}H and ^{13}C NMR spectra were recorded at 400 and 100 MHz, respectively, with TMS as the internal standard. ^{19}F NMR spectra were recorded at 376 MHz with $CFCl_3$ (positive for downfield shifts) as the external standard. High resolution mass data were recorded on a Waters Micromass GCT Preminer instrument. All reactions were monitored by TLC with Huanghai GF_{254} silica gel coated plates. Flash column chromatography was carried out using 300–400 mesh silica gel.

2. General procedure for the trifluoromethylation of terminal alkenes

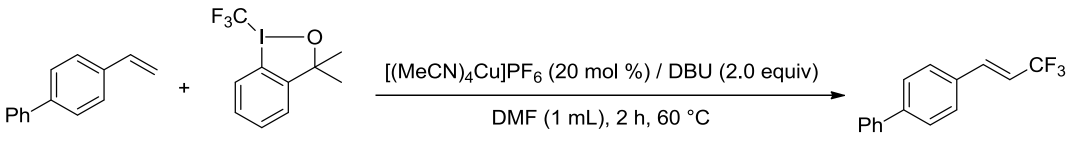

1a Togni's reagent **2a**

Into a solution of 4-vinylbiphenyl (90 mg, 0.5 mmol) in DMF (1 mL) was added Togni's reagent (415 mg, 1.25 mmol) and DBU (152 mg, 1 mmol), followed by $[(MeCN)_4Cu]PF_6$ (37 mg, 0.1 mmol). The reaction mixture was stirred at 60 °C for 2 h. After being cooled to room temperature, the mixture was subjected to column chromatography to afford pure product **2a**.

(*E*)-4-(3,3,3-Trifluoroprop-1-en-1-yl)-1,1'-biphenyl (**2a**)[1]

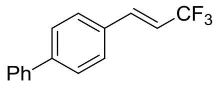

White solid, 90% yield; ^{1}H NMR (400 MHz, $CDCl_3$): δ 7.69 – 7.34 (m, 9H), 7.19 (dq, *J* = 16.0 Hz, 2.0 Hz, 1H), 6.25 (dq, *J* = 16.0 Hz, 6.6 Hz, 1H). ^{19}F NMR (376 MHz, $CDCl_3$): δ –63.24 (dd, *J* = 6.6 Hz, 2.0 Hz, 3F). GC-MS: 248 $[M]^+$.

(*E*)-1-Methoxy-4-(3,3,3-trifluoroprop-1-en-1-yl)benzene (**2b**)[1]

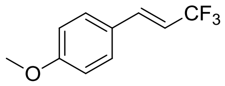

Colorless oil, 91% yield; ^{1}H NMR (400 MHz, $CDCl_3$): δ 7.40 (d, *J* = 8.8 Hz, 2H), 7.09 (dq, *J* = 16.0 Hz, 2.0 Hz, 1H), 6.91 (d, *J* = 8.8 Hz, 2H), 6.06 (dq, *J* = 16.0 Hz, 6.6 Hz, 1H) 3.84 (s, 3H). ^{19}F NMR (376 MHz, $CDCl_3$): δ –62.89 (dd, *J* = 6.6 Hz, 2.0 Hz, 3F). GC-MS: 202 $[M]^+$.

(*E*)-2-Methoxy-6-(3,3,3-trifluoroprop-1-en-1-yl)naphthalene (**2c**) [1]

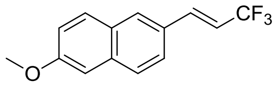

White solid, 88% yield; ^{1}H NMR (400 MHz, $CDCl_3$): δ 7.79 (s, 1H), 7.77 – 7.69 (m, 2H), 7.60 – 7.52 (m, 1H), 7.27 (dq, *J* = 16.0 Hz, 2.0 Hz, 1H), 7.21 – 7.10 (m, 2H), 6.26 (dq, *J* = 16.0 Hz, 6.5 Hz, 1H), 3.94 (s, 3H). ^{19}F NMR (376 MHz, $CDCl_3$): δ –63.00 (dd, *J* = 6.5 Hz, 2.0 Hz, 3F). GC-MS: 252 $[M]^+$.

(*E*)-1-(*tert*-Butyl)-4-(3,3,3-trifluoroprop-1-en-1-yl)benzene (**2d**) [1]

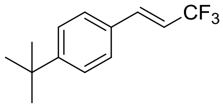

Colorless oil, 88% yield; ^{1}H NMR (400 MHz, $CDCl_3$): δ 7.49 – 7.31 (m, 4H), 7.13 (dq, *J* = 16.1 Hz, 2.1 Hz, 1H), 6.16 (dq, *J* = 16.1 Hz, 6.6 Hz, 1H), 1.33 (s, 9H). ^{19}F NMR (376 MHz, $CDCl_3$): δ –63.17 (dd, *J* = 6.6 Hz, 2.1 Hz, 3F). GC-MS: 228 $[M]^+$.

(*E*)-1-Methyl-4-(3,3,3-trifluoroprop-1-en-1-yl)benzene (**2e**) [1]

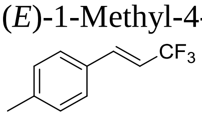

White solid, 92% yield; ^{1}H NMR (400 MHz, $CDCl_3$): δ 7.35 (d, *J* = 8.0 Hz, 2H), 7.20 (d, *J* = 8.0 Hz, 2H), 7.12 (dq, *J* = 16.1 Hz, 2.0 Hz, 1H), 6.16 (dq, *J* = 16.1 Hz, 6.6 Hz, 1H), 2.38 (s, 3H). ^{19}F NMR (376 MHz, $CDCl_3$): δ –63.16 (dd, *J* = 6.6 Hz, 2.0 Hz, 3F). GC-MS: 186 $[M]^+$.

(*E*)-2-(3,3,3-Trifluoroprop-1-en-1-yl)naphthalene (**2f**) [1]

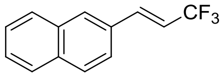

White solid, 92% yield; ^{1}H NMR (400 MHz, $CDCl_3$): δ 7.90 – 7.75 (m, 4H), 7.60 (d, *J* = 8.8 Hz, 1H), 7.57 – 7.45 (m, 2H), 7.32 (dd, *J* = 16.0 Hz, 1.5 Hz, 1H), 6.16 (dq, *J* = 16.0 Hz, 6.6 Hz, 1H), 2.38 (s, 3H). ^{19}F NMR (376 MHz, $CDCl_3$): δ –63.20 (dd, *J* = 6.6 Hz, 1.5 Hz, 3F). GC-MS: 222 $[M]^+$.

(*E*)-1-Bromo-2-(3,3,3-trifluoroprop-1-en-1-yl)benzene (**2g**) [2]

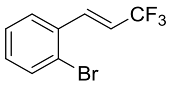

Colorless oil, 93% yield; ^{1}H NMR (400 MHz, $CDCl_3$): δ 7.66 – 7.47 (m, 3H), 7.39 – 7.29 (m, 1H), 7.25 – 7.20 (m, 1H), 6.16 (dq, *J* = 16.0 Hz, 6.4 Hz, 1H). ^{19}F NMR (376 MHz, $CDCl_3$): δ –63.67 (dd, *J* = 6.4 Hz, 2.1 Hz, 3F). GC-MS: 250 $[M]^+$.

(*E*)-1-Bromo-3-(3,3,3-trifluoroprop-1-en-1-yl)benzene (**2h**) [3]

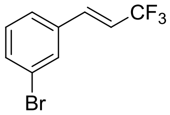

Colorless oil, 92% yield; ^{1}H NMR (400 MHz, $CDCl_3$): δ 7.61(t, *J* = 1.6 Hz, 1H), 7.51 (dt, *J* = 7.8 Hz, 1.6 Hz, 1H), 7.38 (dt, *J* = 7.8 Hz, 1.6 Hz, 1H), 7.27 (t, *J* = 7.8 Hz, 1H),

7.08 (dq, J = 16.1 Hz, 2.1 Hz, 1H), 6.21 (dq, J = 16.1 Hz, 6.4 Hz, 1H). ^{19}F NMR (376 MHz, $CDCl_3$): δ –63.64 (dd, J = 6.4 Hz, 2.1 Hz, 3F). GC-MS: 250 $[M]^+$.

(*E*)-1-Bromo-4-(3,3,3-trifluoroprop-1-en-1-yl)benzene (**2i**) [4]

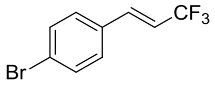

Colorless oil, 93% yield; ^{1}H NMR (400 MHz, $CDCl_3$): δ 7.53 (d, J = 8.4 Hz, 2H), 7.32 (d, J = 8.4 Hz, 2H), 7.09 (dq, J = 16.1 Hz, 2.0 Hz, 1H), 6.20 (dq, J = 16.1 Hz, 6.5 Hz, 1H). ^{19}F NMR (376 MHz, $CDCl_3$): δ –63.52 (dd, J = 6.5 Hz, 2.1 Hz, 3F). GC-MS: 250 $[M]^+$.

(*E*)-1-Chloro-4-(3,3,3-trifluoroprop-1-en-1-yl)benzene (**2j**) [4]

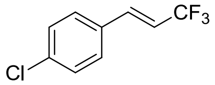

Colorless oil, 86% yield; ^{1}H NMR (400 MHz, $CDCl_3$): δ 7.44 – 7.31 (m, 4H), 7.11 (dq, J = 16.0 Hz, 2.0 Hz, 1H), 6.18 (dq, J = 16.0 Hz, 6.4 Hz, 1H). ^{19}F NMR (376 MHz, $CDCl_3$): δ –63.48 (dd, J = 6.4 Hz, 2.0 Hz, 3F). GC-MS: 206 $[M]^+$.

(*E*)-1-Fluoro-3-(3,3,3-trifluoroprop-1-en-1-yl)benzene (**2k**) [5]

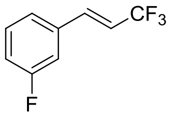

Colorless oil, 63% yield; ^{1}H NMR (400 MHz, $CDCl_3$): δ 7.41 – 7.32 (m, 1H), 7.25 – 7.20 (m, 1H), 7.19 – 7.05 (m, 3H), 6.21 (dq, J = 16.0 Hz, 6.4 Hz, 1H). ^{19}F NMR (376 MHz, $CDCl_3$): δ –63.63 (dd, J = 6.4 Hz, 2.0 Hz, 3F), -112.41 (td, J = 9.0 Hz, 5.9 Hz, 1F). GC-MS: 190 $[M]^+$.

(*E*)-3-(3,3,3-Trifluoroprop-1-en-1-yl)benzaldehyde (**2l**)

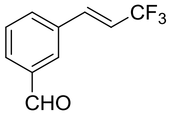

Colorless oil, 84% yield; ^{1}H NMR (400 MHz, $CDCl_3$): δ 10.05 (s, 1H), 7.97 (s, 1H), 7.90 (d, J = 7.6 Hz, 1H), 7.71 (d, J = 7.6 Hz, 1H), 7.59 (t, J = 7.6 Hz, 1H), 7.22 (dq, J = 16.0 Hz, 2.0 Hz, 1H), 6.32 (dq, J = 16.0 Hz, 6.4 Hz, 1H). ^{19}F NMR (376 MHz, $CDCl_3$): δ –63.70 (dd, J = 6.4 Hz, 2.0 Hz, 3F). ^{13}C NMR (100 MHz, $CDCl_3$): δ 191.52, 136.90, 136.26 (q, J = 6.8 Hz), 134.38, 133.08, 131.10, 129.70, 128.13, 123.18 (q, J = 269.2 Hz), 117.74 (q, J = 34.2 Hz). IR (KBr): 3069, 2928, 2835, 2736, 1702, 1669, 1602, 1310, 1277, 1130, 972, 795, 685, 651, 579 cm^{-1}. GC-MS: 200 $[M]^+$. HRMS: Calculated for $C_{10}H_7OF_3$: 200.0449; Found: 200.0448.

(*E*)-1-Nitro-4-(3,3,3-trifluoroprop-1-en-1-yl)benzene (**2m**) [6]

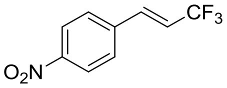

White solid, 76% yield; ^{1}H NMR (400 MHz, $CDCl_3$): δ 8.27 (d, *J* = 8.8 Hz, 2H), 7.63 (d, *J* = 8.8 Hz, 2H), 7.22 (dq, *J* = 16.1 Hz, 2.0 Hz, 1H), 6.36 (dq, *J* = 16.1 Hz, 6.4 Hz, 1H). ^{19}F NMR (376 MHz, $CDCl_3$): δ –64.02 (dd, *J* = 6.4 Hz, 2.0 Hz, 3F). GC-MS: 217 $[M]^+$.

(*E*)-3-(3,3,3-Trifluoroprop-1-en-1-yl)benzo[*b*]thiophene (**2n**)

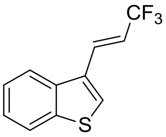

Colorless oil, 89% yield; ^{1}H NMR (400 MHz, $CDCl_3$): δ 7.99 – 7.82 (m, 2H), 7.67 (s, 1H), 7.52 – 7.37 (m, 3H), 6.31 (dq, *J* = 16.0 Hz, 6.4 Hz, 1H). ^{19}F NMR (376 MHz, $CDCl_3$): δ –63.42 (dd, *J* = 6.4 Hz, 2.0 Hz, 3F). ^{13}C NMR (100 MHz, $CDCl_3$): δ 140.46, 136.90, 130.25, 129.75 (q, *J* = 7.1 Hz), 126.81 (q, *J* = 1.2 Hz), 125.04, 124.89, 123.50 (q, *J* = 268.9 Hz), 123.06, 121.67, 116.75 (q, *J* = 33.8 Hz). IR (KBr): 3072, 1663, 1511, 1427, 1369, 1298, 1288, 1272, 1128, 964, 891, 757, 730 cm^{-1}. GC-MS: 228 $[M]^+$. HRMS: Calculated for $C_{11}H_7SF_3$: 228.0221; Found:228.0217.

(*E*)-2-Bromo-6-(3,3,3-trifluoroprop-1-en-1-yl)pyridine (**2o**)

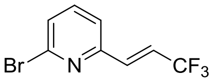

Colorless oil, 70% yield; ^{1}H NMR (400 MHz, $CDCl_3$): δ 7.57 (t, *J* = 7.7 Hz, 1H), 7.46 (d, *J* = 7.7 Hz, 1H), 7.28 (d, *J* = 7.7 Hz, 1H), 7.08 (dq, *J* = 15.6 Hz, 2.0 Hz, 1H), 6.86 (dq, *J* = 15.6 Hz, 6.8 Hz, 1H). ^{19}F NMR (376 MHz, $CDCl_3$): δ –64.08 (dd, *J* = 6.8 Hz, 2.0 Hz, 3F). ^{13}C NMR (100 MHz, $CDCl_3$): δ 152.79, 142.55, 139.07, 134.90 (q, *J* = 6.7 Hz), 128.70, 123.17 (q, *J* = 269.2 Hz), 122.65, 121.52 (q, *J* = 34.4 Hz). IR (KBr): 3069, 2927, 2854, 1672, 1580, 1557, 1435, 1412, 1331, 1307, 1269, 1163, 1136, 985, 904, 786, 729, 661, 586 cm^{-1}. GC-MS: 251 $[M]^+$. HRMS: Calculated for $C_8H_5BrNF_3$: 250.9557; Found: 250.9559.

References

1. Lin, H.; Dong, X.; Li, Y.; Shen, Q.; Lu, L. *Eur. J. Org. Chem.* **2012**, 4675-4679.
2. Hafner, A.; Bräse, S. *Adv. Synth. Catal.* **2011**, *353*, 3044-3048.
3. Fialkov, Yu. A.; Yagupol'skii, L. M. *Ukrainskii Khimicheskii Zhurnal (Russian Edition)* **1966**, *32*, 996-1002.
4. Yasu, Y.; Koike, T.; Akita, M. *Chem. Commun.* **2013**, *49*, 2037-2039.
5. Fuchikami, T.; Yatabe, M.; Ojima, I. *Synthesis* **1981**, *1981*, 365-366.
6. Landge, S. M.; Borkin, D. A.; Török, B. *Tetrahedron Lett.* **2007**, *48*, 6372-6376.

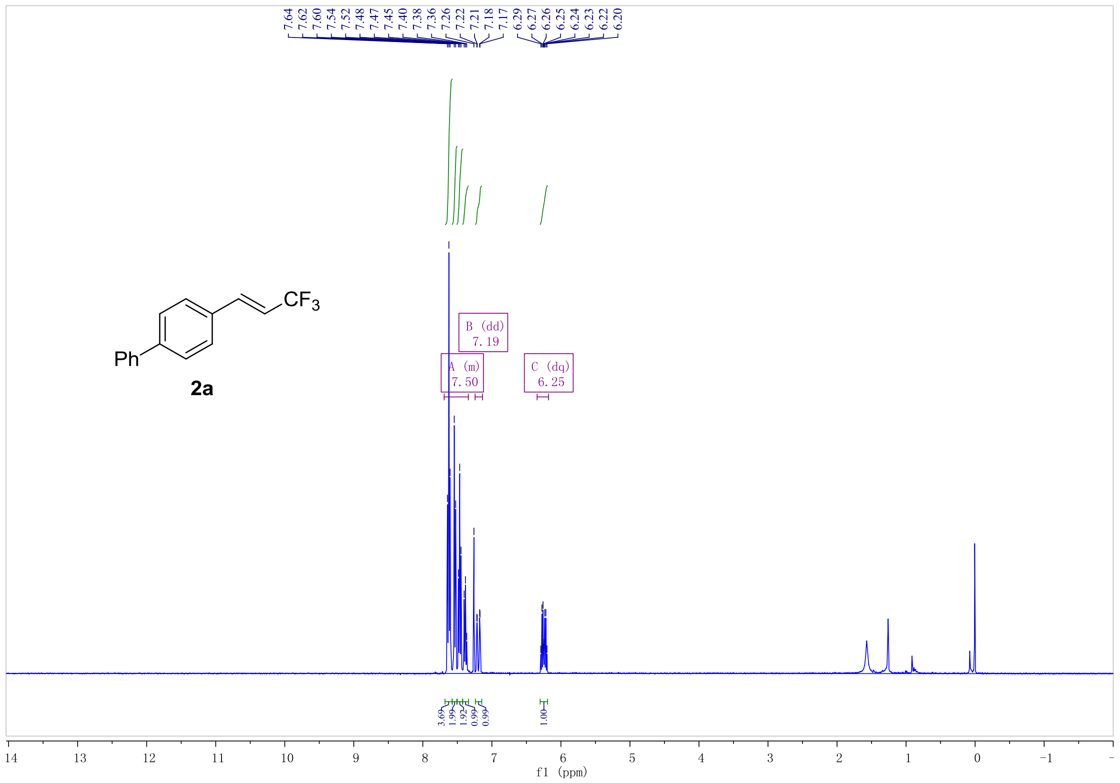

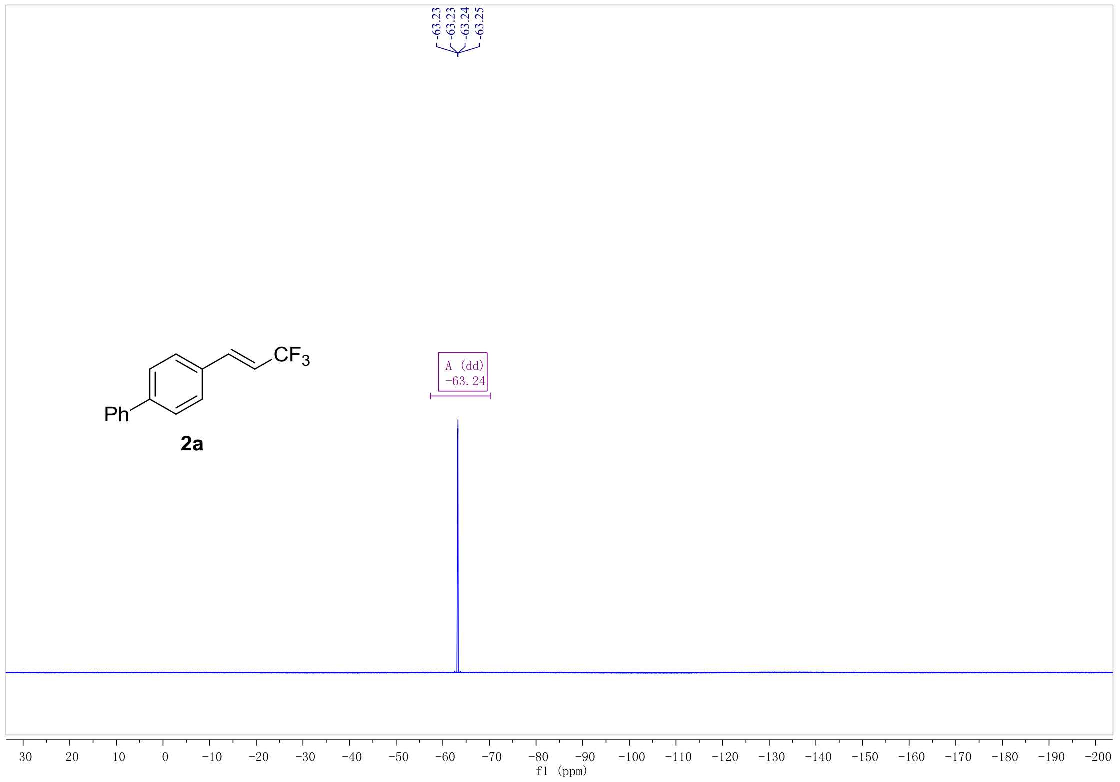

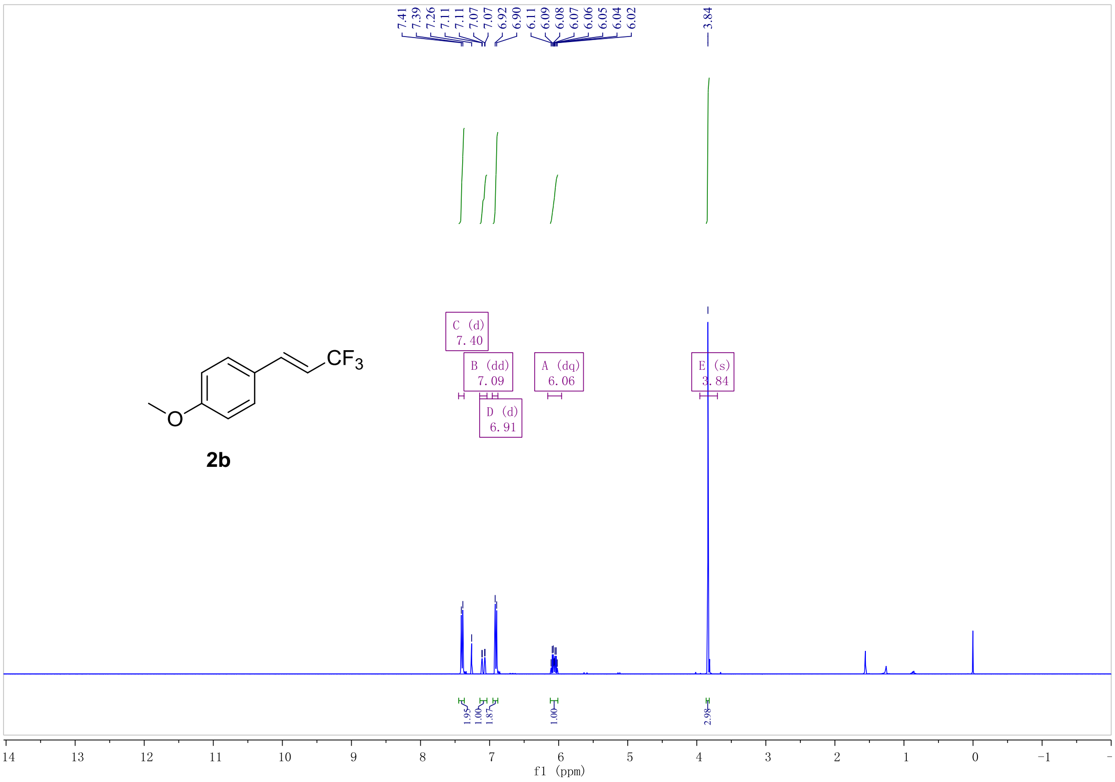

7.41
7.39
7.26
7.11
7.11
7.07
7.07
6.92
6.90
6.11
6.09
6.08
6.07
6.06
6.05
6.04
6.02
3.84
C (d)
7.40
B (dd)
7.09
D (d)
6.91
A (dq)
6.06
E (s)
3.84
CF3
O
2b
1.95
1.00
1.87
1.00
2.98
f1 (ppm)

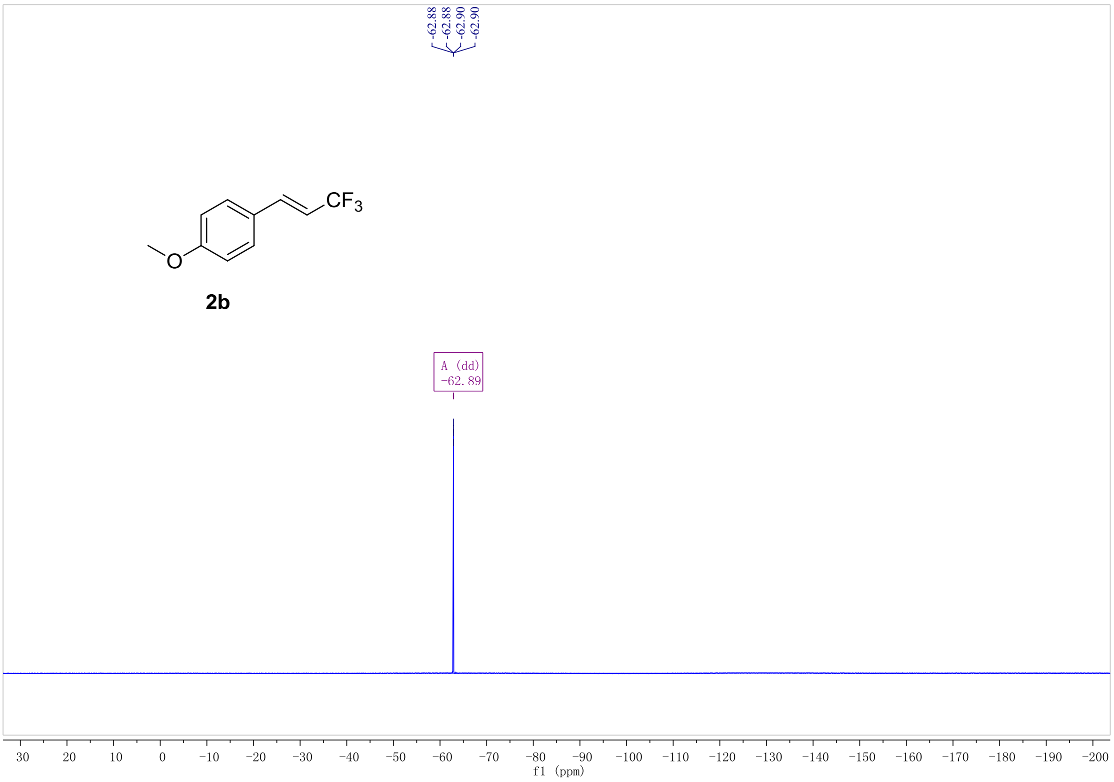

-62.88
-62.88
-62.90
-62.90
CF3
O
2b
A (dd)
-62.89
f1 (ppm)

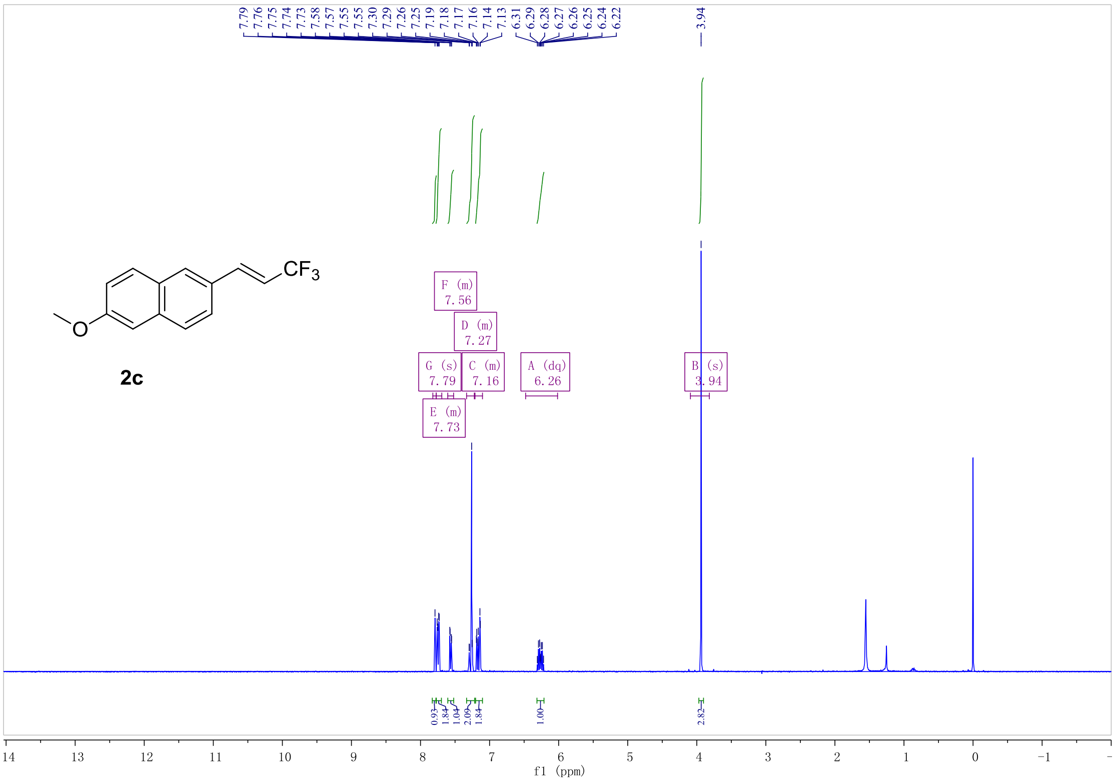
CF3
O
2c
G (s) 7.79
E (m) 7.73
F (m) 7.56
D (m) 7.27
C (m) 7.16
A (dq) 6.26
B (s) 3.94
f1 (ppm)

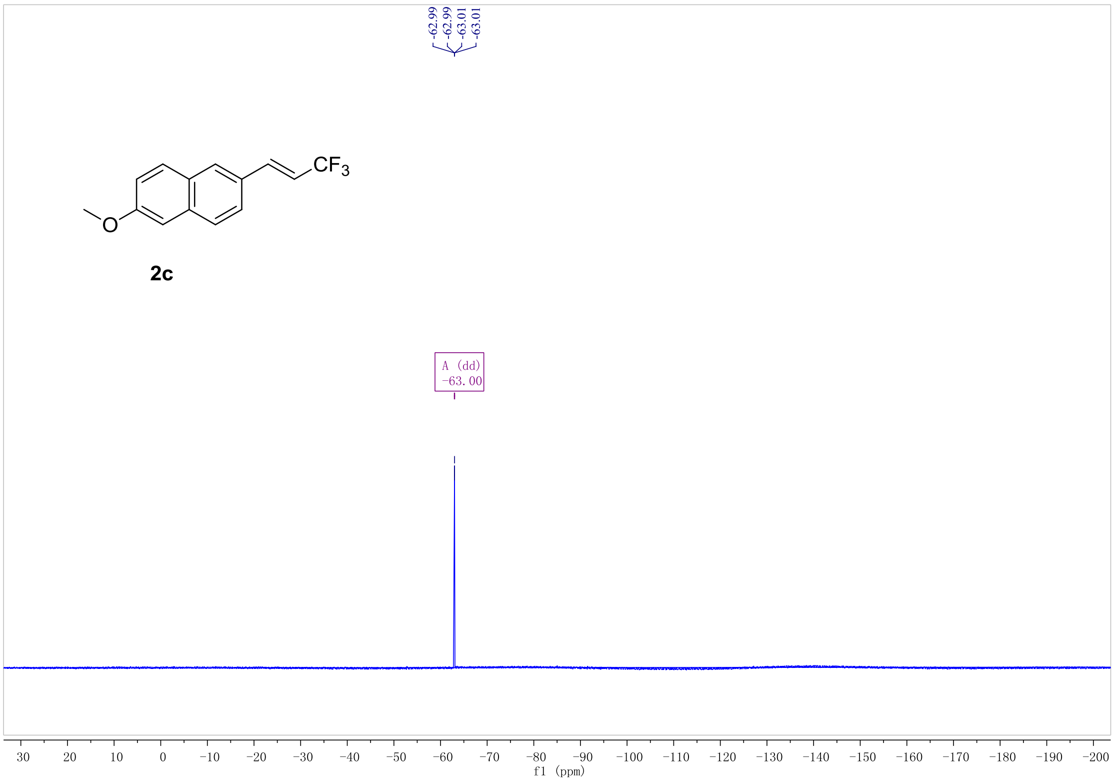
-62.99
-62.99
-63.01
-63.01
CF3
O
2c
A (dd) -63.00
f1 (ppm)

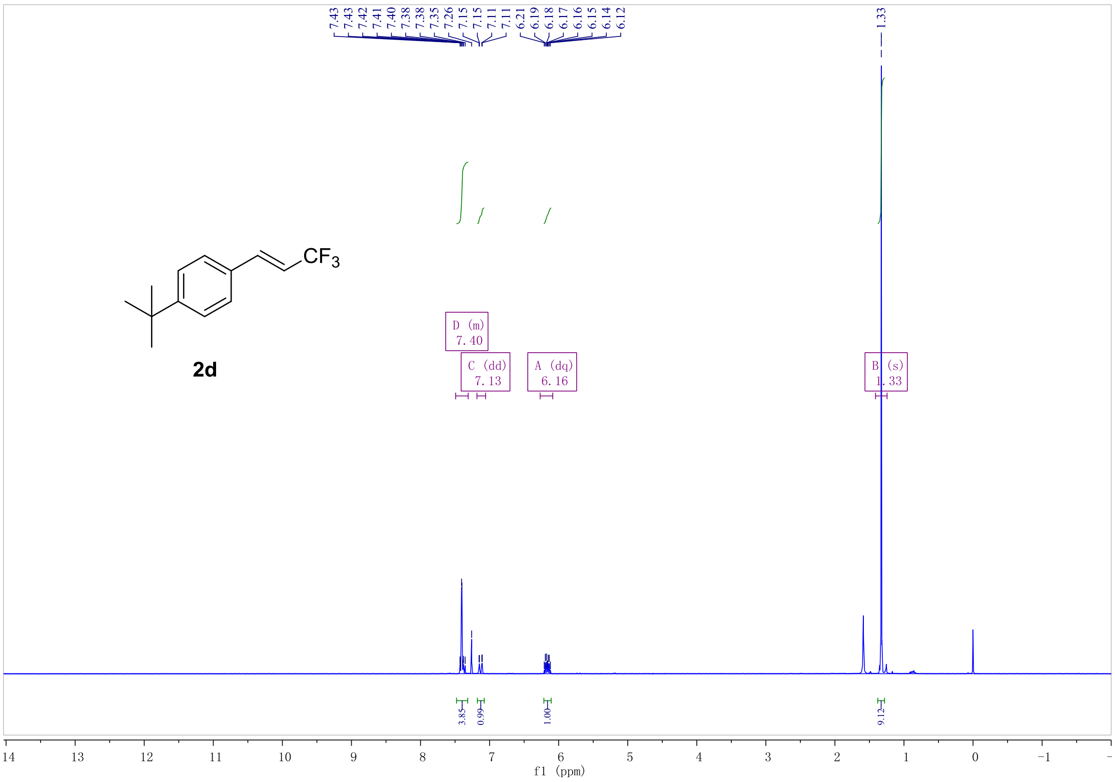

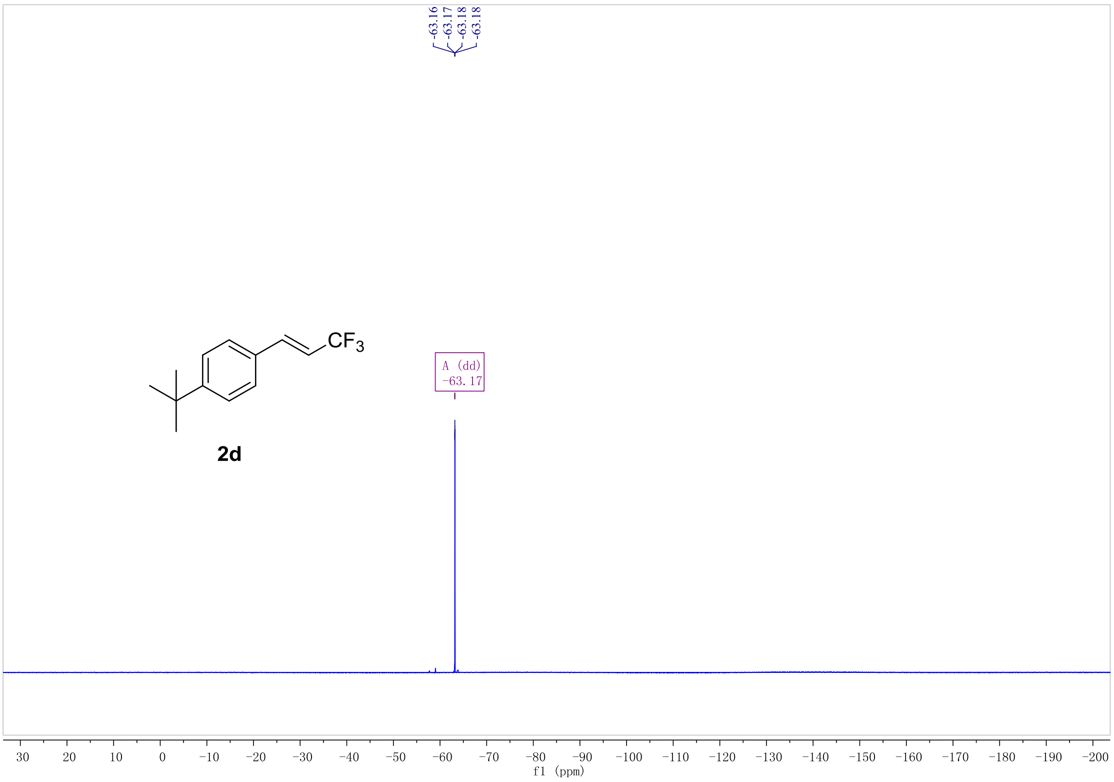

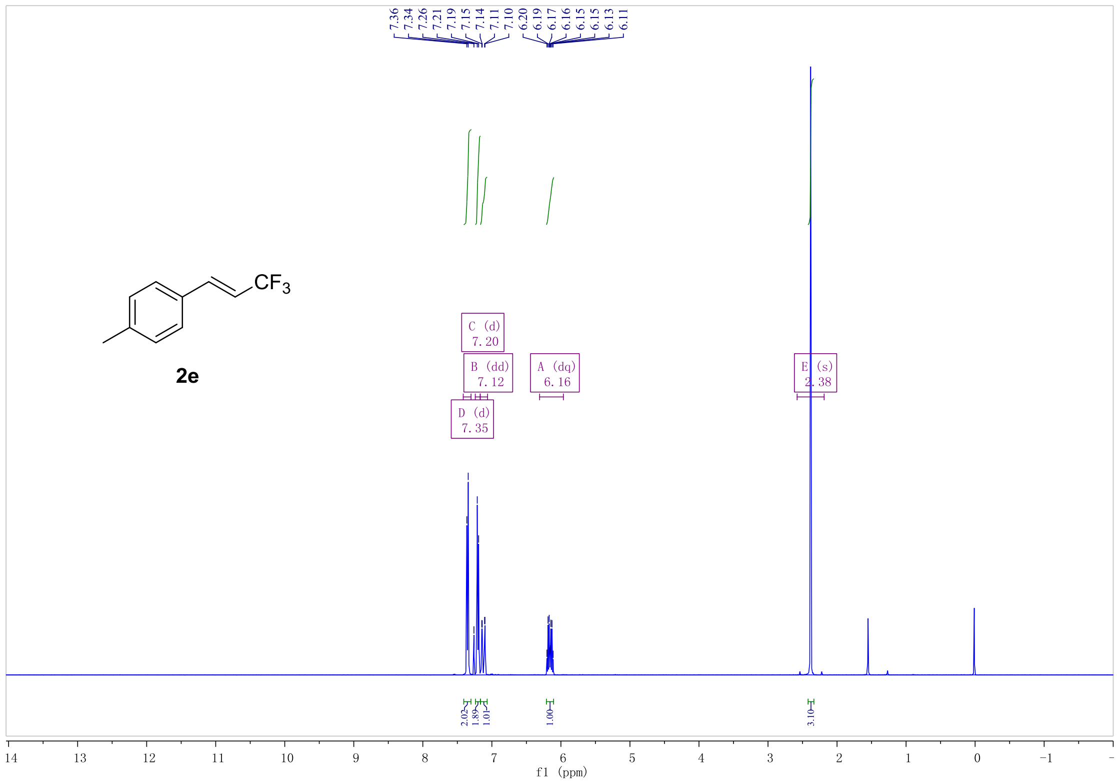
7.36 7.34 7.26 7.21 7.19 7.15 7.14 7.11 7.10 6.20 6.19 6.17 6.16 6.15 6.13 6.11
CF3
2e
C (d) 7.20
B (dd) 7.12
A (dq) 6.16
D (d) 7.35
E (s) 2.38
2.03 1.89 1.01 1.00 3.10
f1 (ppm)

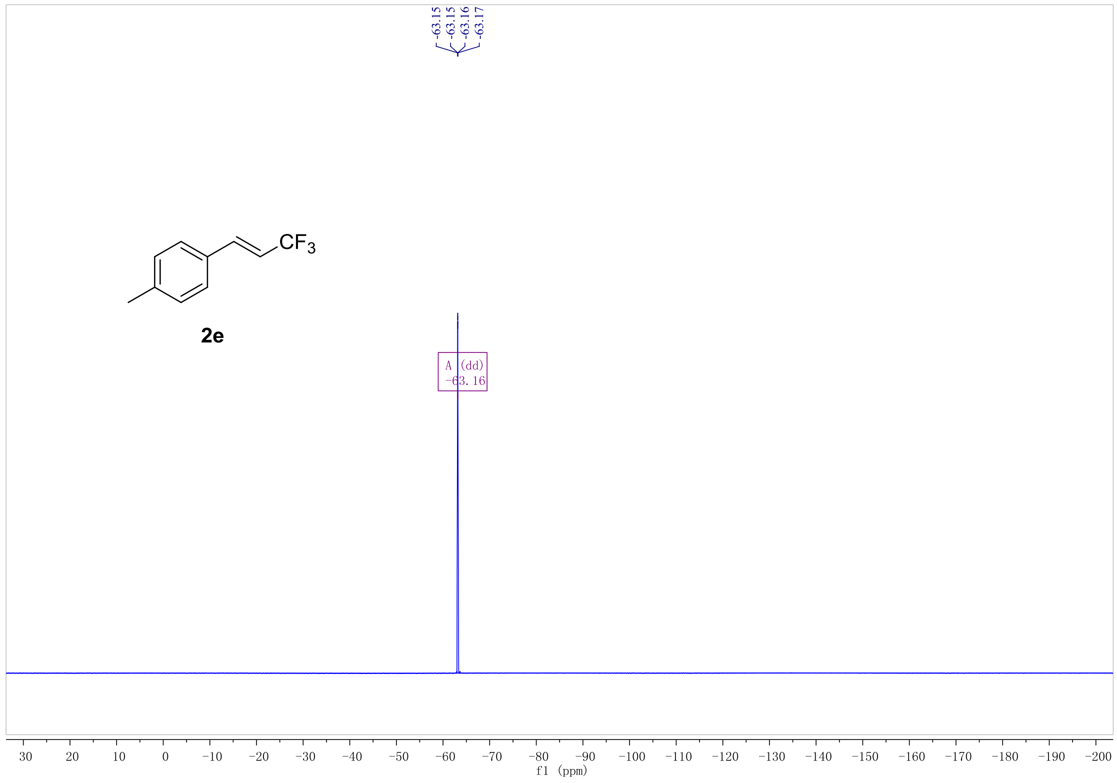
-63.15 -63.15 -63.16 -63.17
CF3
2e
A (dd) -63.16
f1 (ppm)

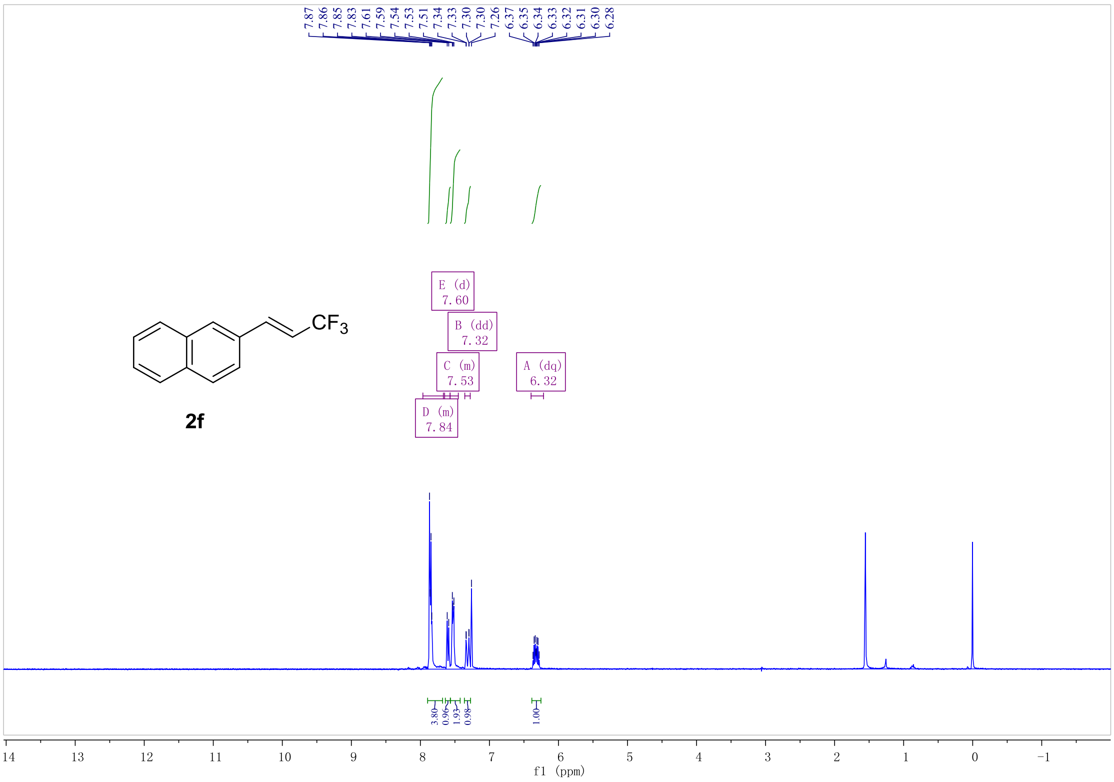
2f
E (d) 7.60
B (dd) 7.32
C (m) 7.53
A (dq) 6.32
D (m) 7.84
f1 (ppm)

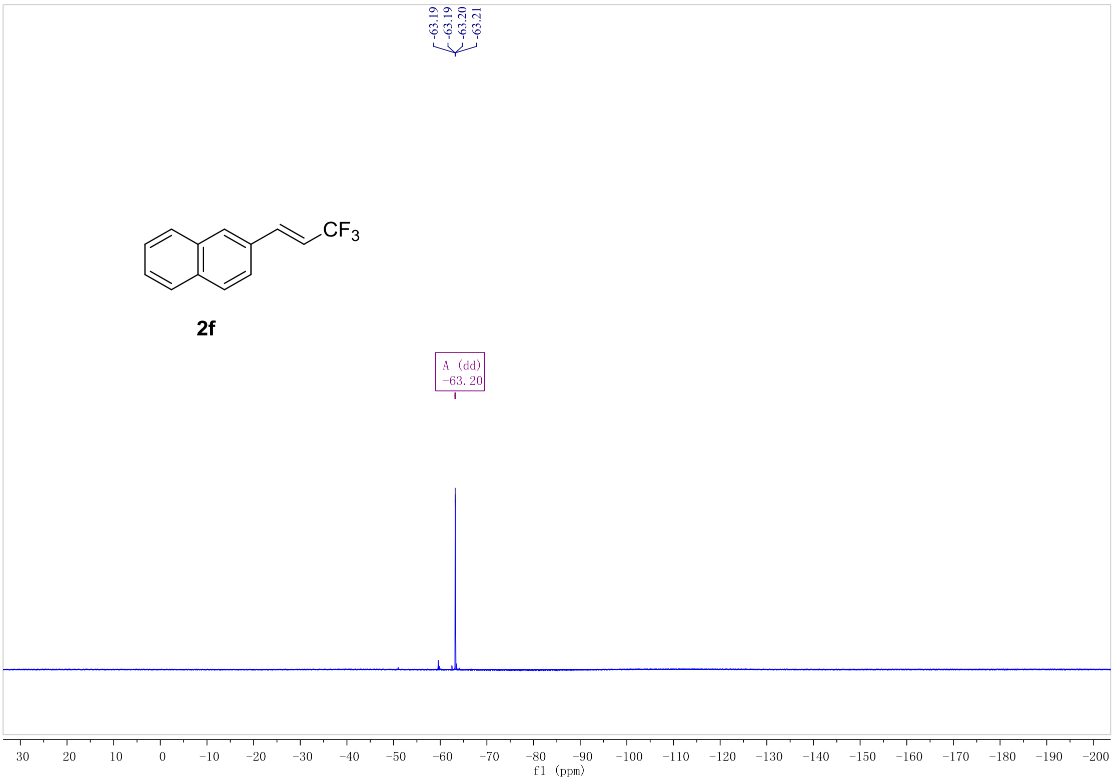
2f
A (dd) -63.20
f1 (ppm)

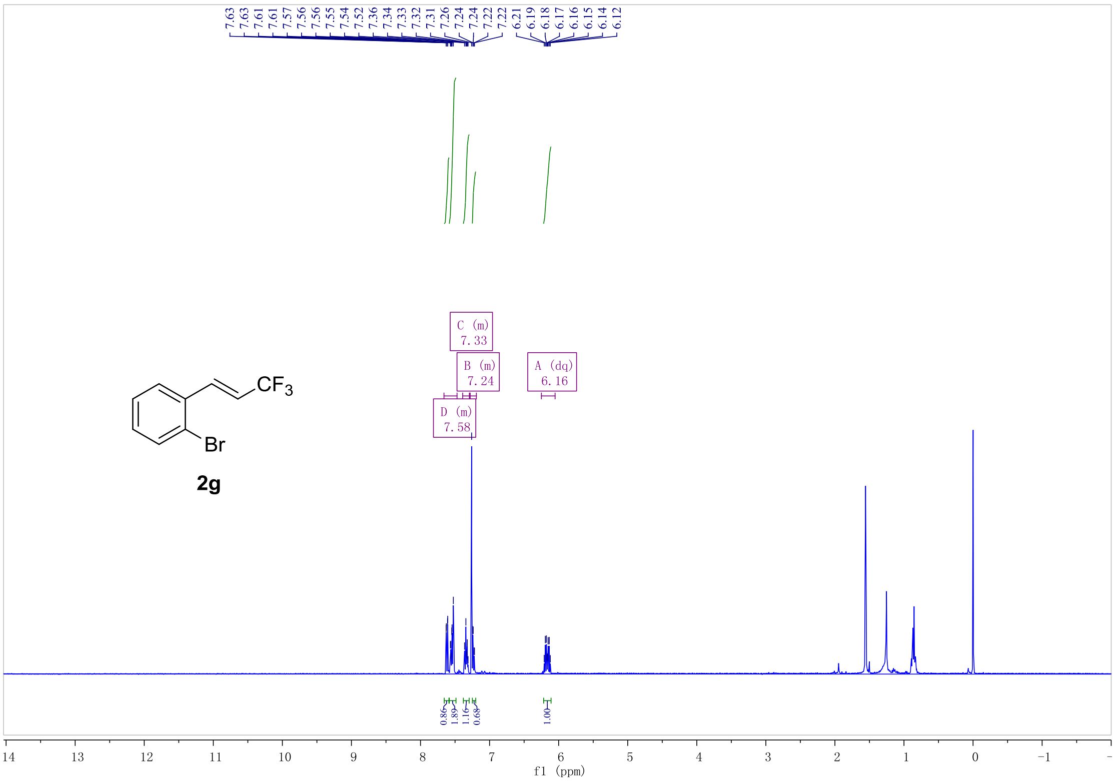
7.63
7.63
7.61
7.61
7.57
7.56
7.56
7.55
7.54
7.52
7.36
7.34
7.33
7.32
7.31
7.26
7.24
7.24
7.22
7.22
6.21
6.19
6.18
6.17
6.16
6.15
6.14
6.12
CF3
Br
2g
C (m)
7.33
B (m)
7.24
A (dq)
6.16
D (m)
7.58
0.86
1.89
1.16
0.68
1.00
14 13 12 11 10 9 8 7 6 5 4 3 2 1 0 -1
f1 (ppm)

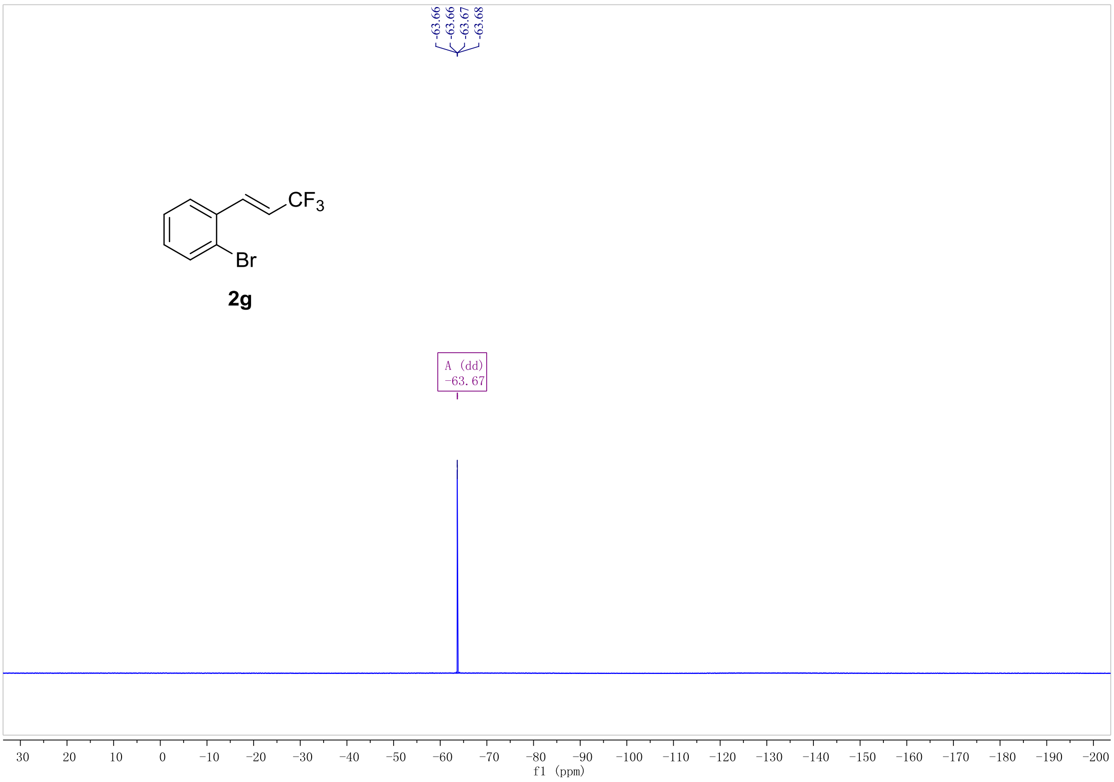
-63.66
-63.66
-63.67
-63.68
CF3
Br
2g
A (dd)
-63.67
30 20 10 0 -10 -20 -30 -40 -50 -60 -70 -80 -90 -100 -110 -120 -130 -140 -150 -160 -170 -180 -190 -200
f1 (ppm)

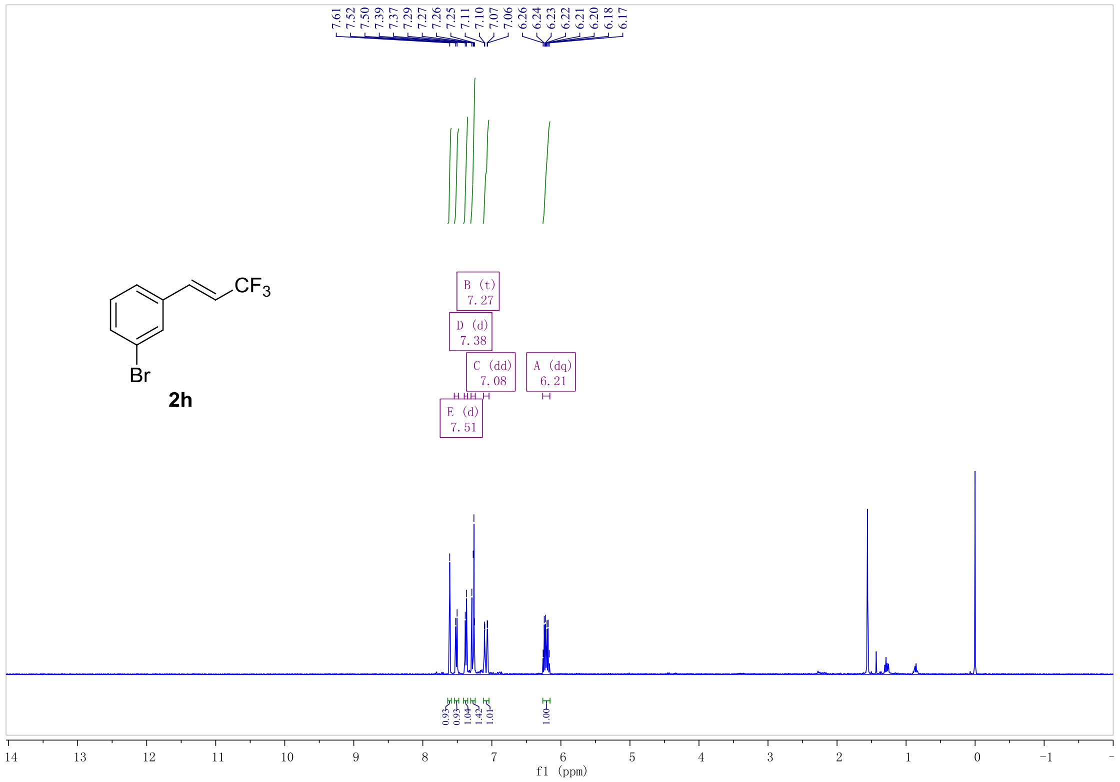
7.61
7.52
7.50
7.39
7.37
7.29
7.27
7.26
7.25
7.11
7.10
7.07
7.06
6.26
6.24
6.23
6.22
6.21
6.20
6.18
6.17
CF3
Br
2h
B (t) 7.27
D (d) 7.38
C (dd) 7.08
A (dq) 6.21
E (d) 7.51
0.93
0.93
1.04
1.42
1.01
1.00
f1 (ppm)

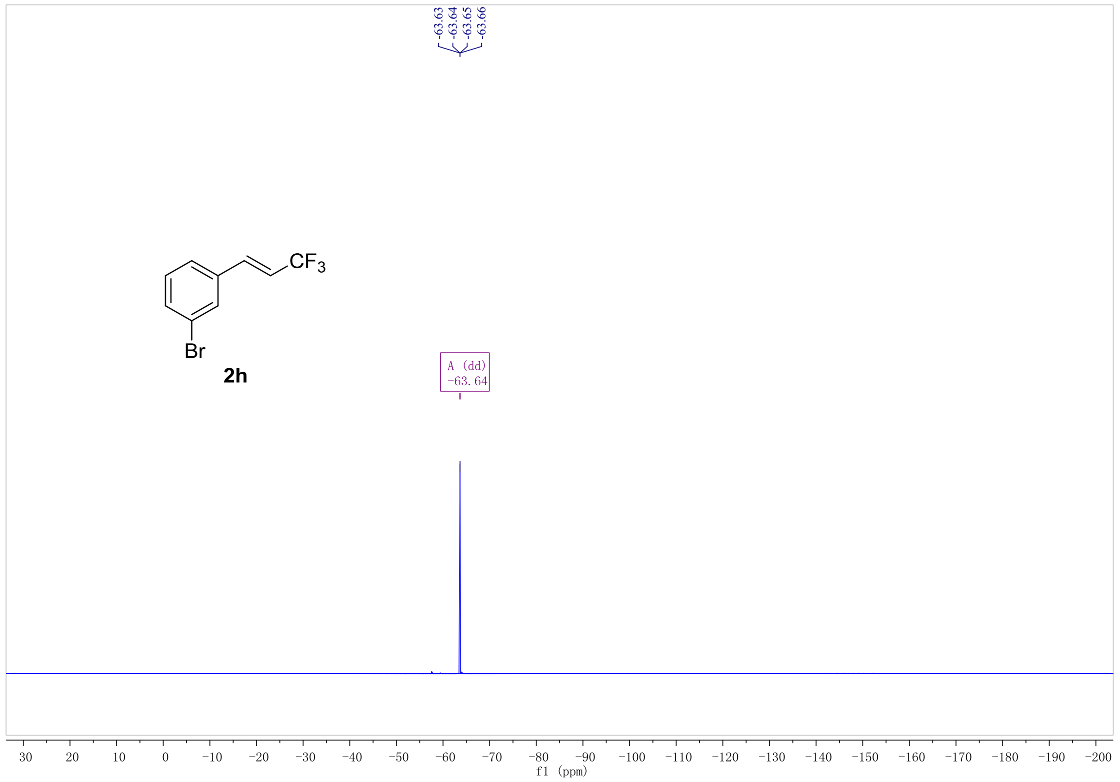
-63.63
-63.64
-63.65
-63.66
CF3
Br
2h
A (dd) -63.64
f1 (ppm)

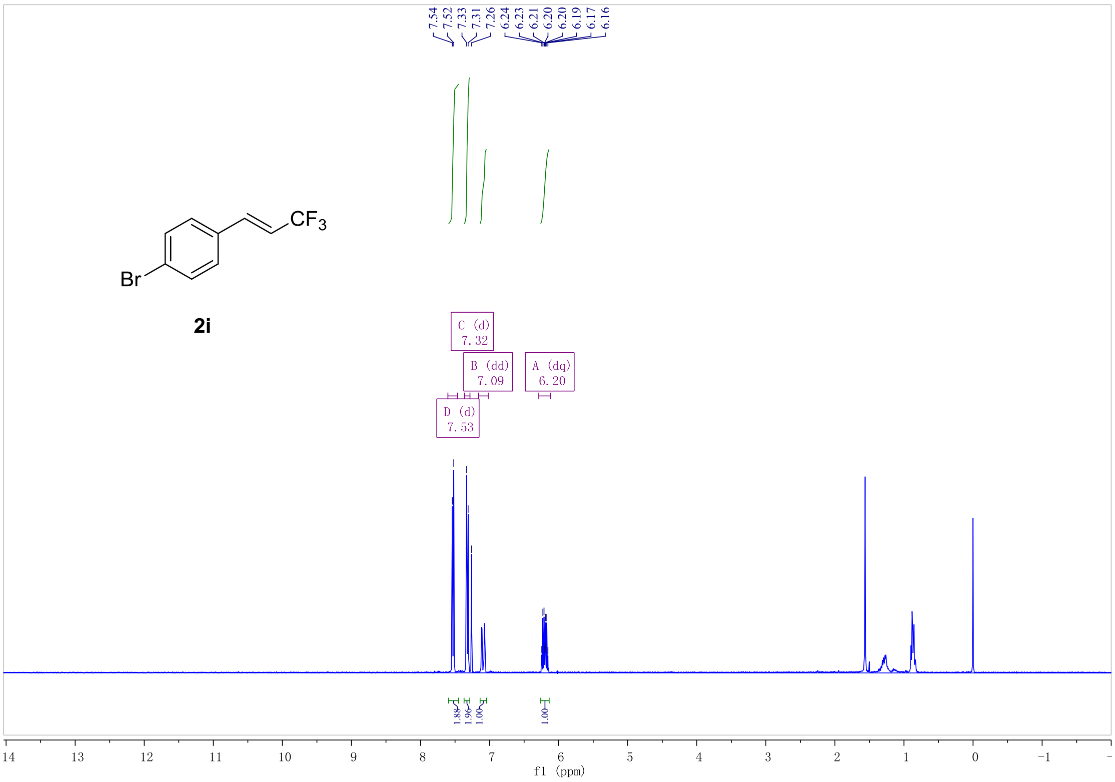
7.54
7.52
7.33
7.31
7.26
6.24
6.23
6.21
6.20
6.20
6.19
6.17
6.16
CF3
Br
2i
C (d)
7.32
B (dd)
7.09
A (dq)
6.20
D (d)
7.53
1.88
1.96
1.00
1.00
14
13
12
11
10
9
8
7
6
5
4
3
2
1
0
-1
f1 (ppm)

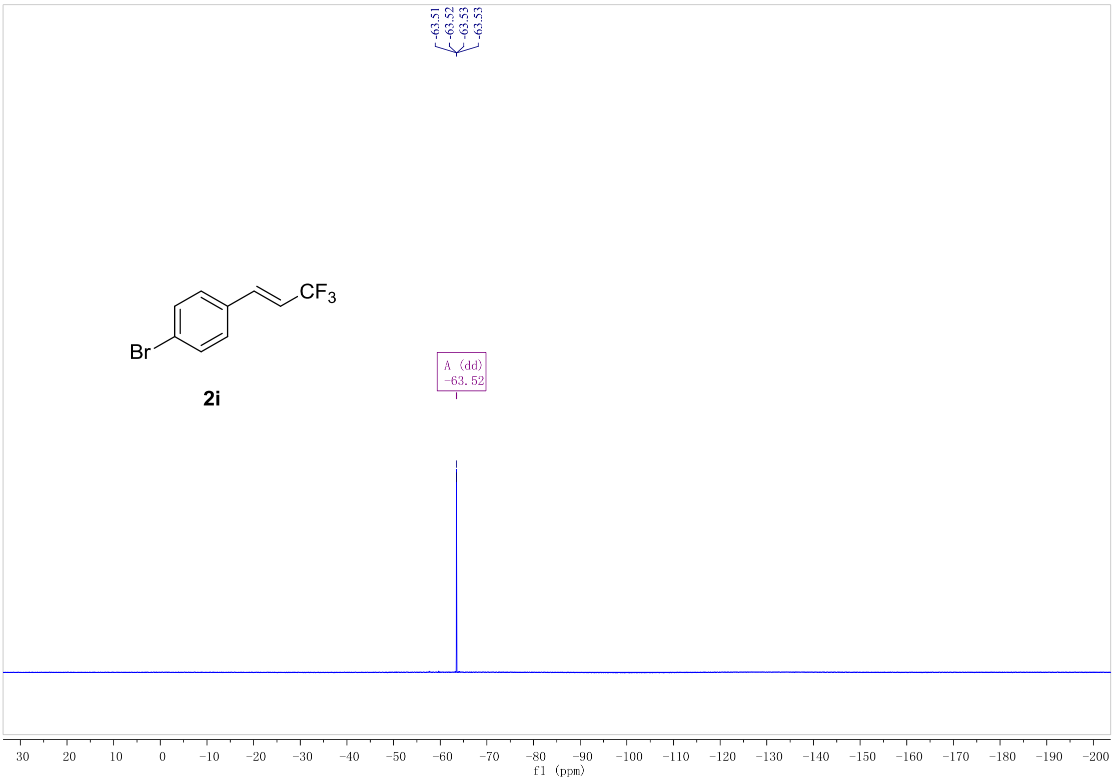
-63.51
-63.52
-63.53
-63.53
CF3
Br
2i
A (dd)
-63.52
30
20
10
0
-10
-20
-30
-40
-50
-60
-70
-80
-90
-100
-110
-120
-130
-140
-150
-160
-170
-180
-190
-200
f1 (ppm)

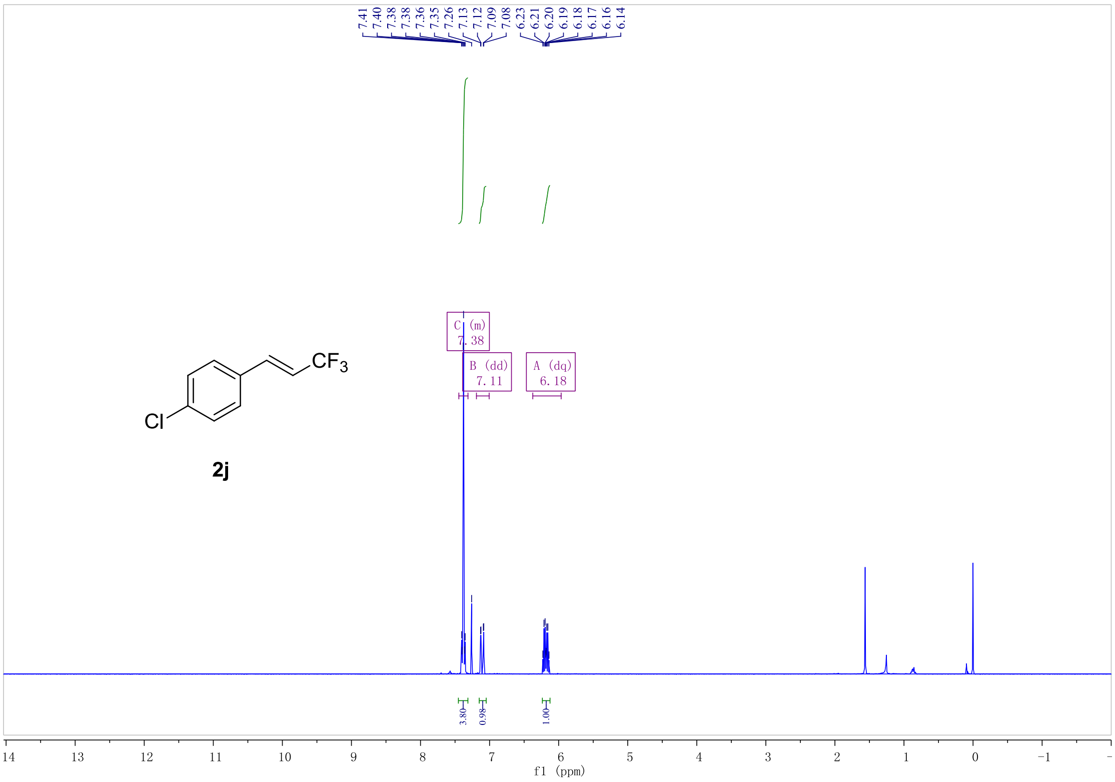
7.41
7.40
7.38
7.38
7.36
7.35
7.26
7.13
7.12
7.09
7.08
6.23
6.21
6.20
6.19
6.18
6.17
6.16
6.14
C (m)
7.38
B (dd)
7.11
A (dq)
6.18
CF3
Cl
2j
3.80
0.98
1.00
f1 (ppm)

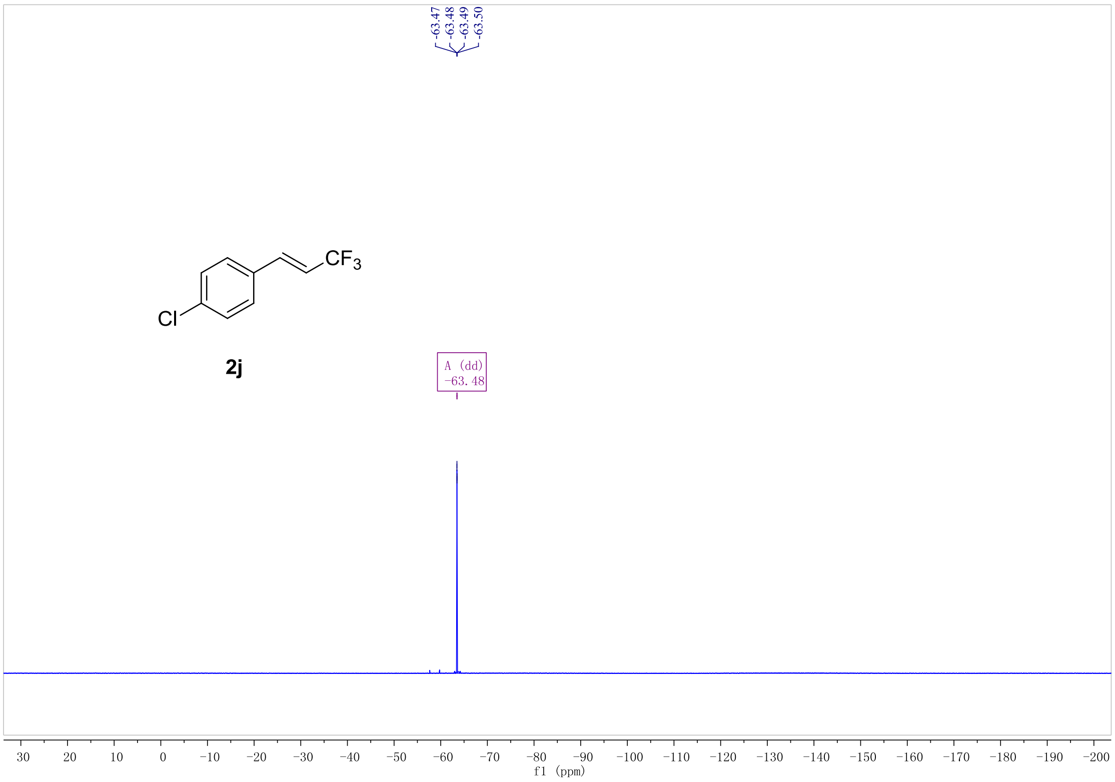
-63.47
-63.48
-63.49
-63.50
CF3
Cl
2j
A (dd)
-63.48
f1 (ppm)

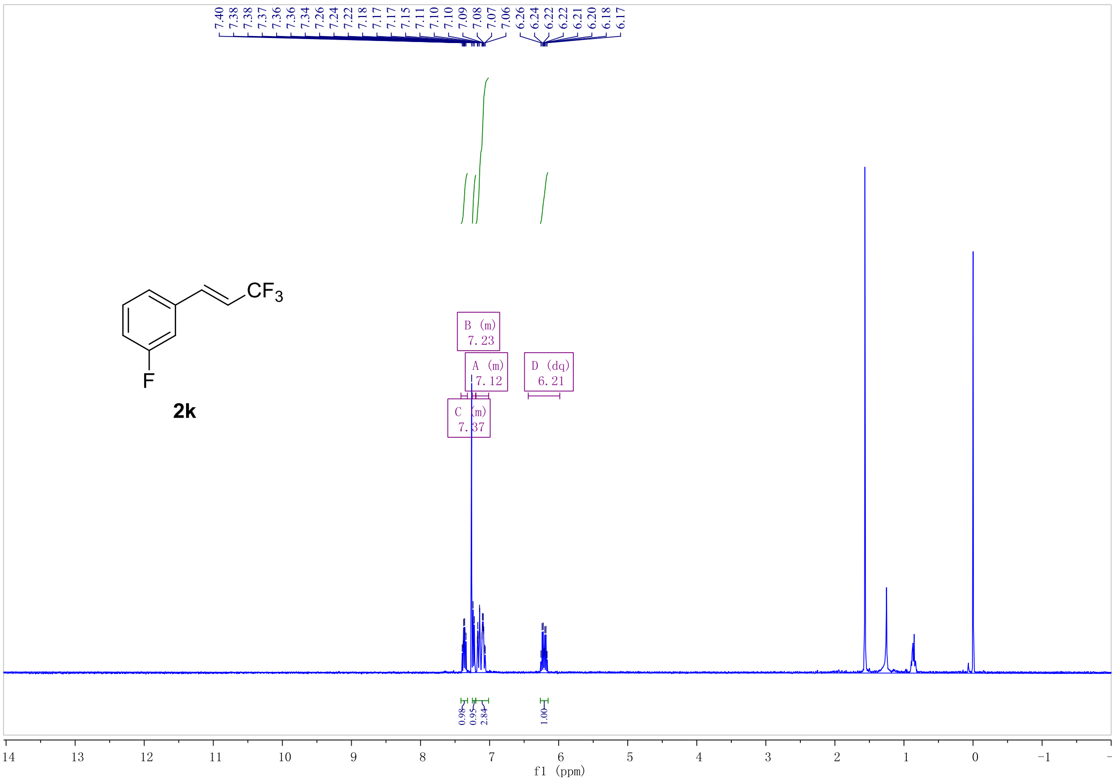

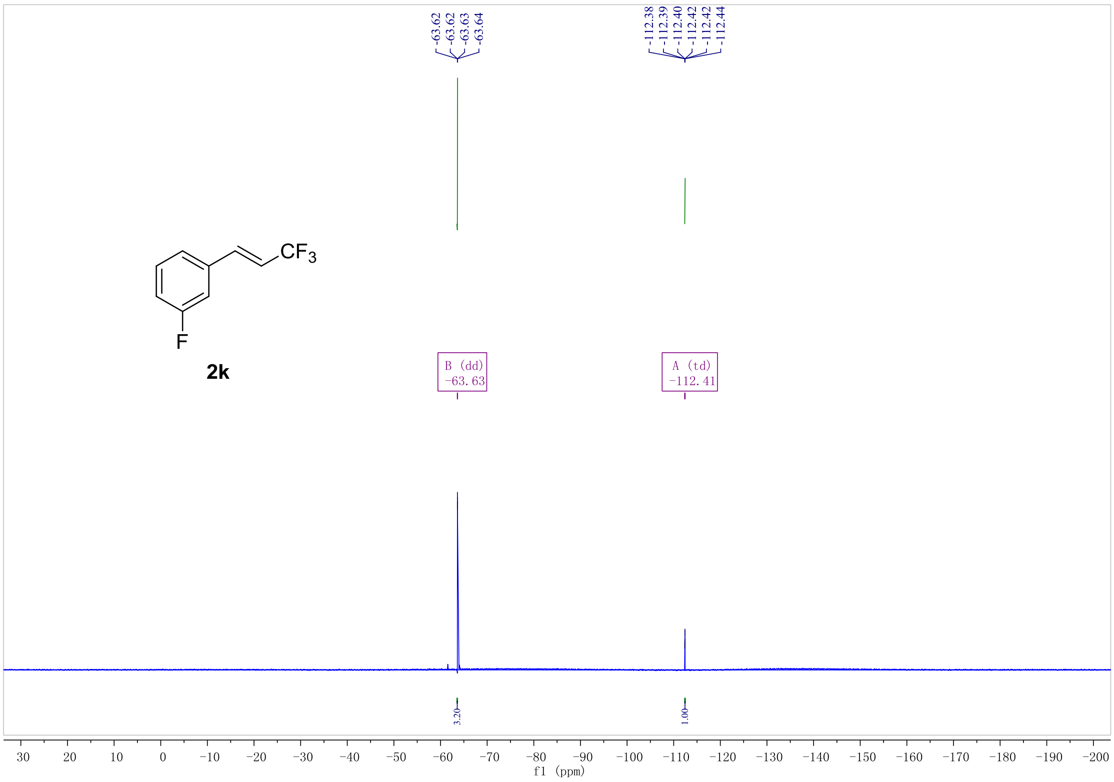

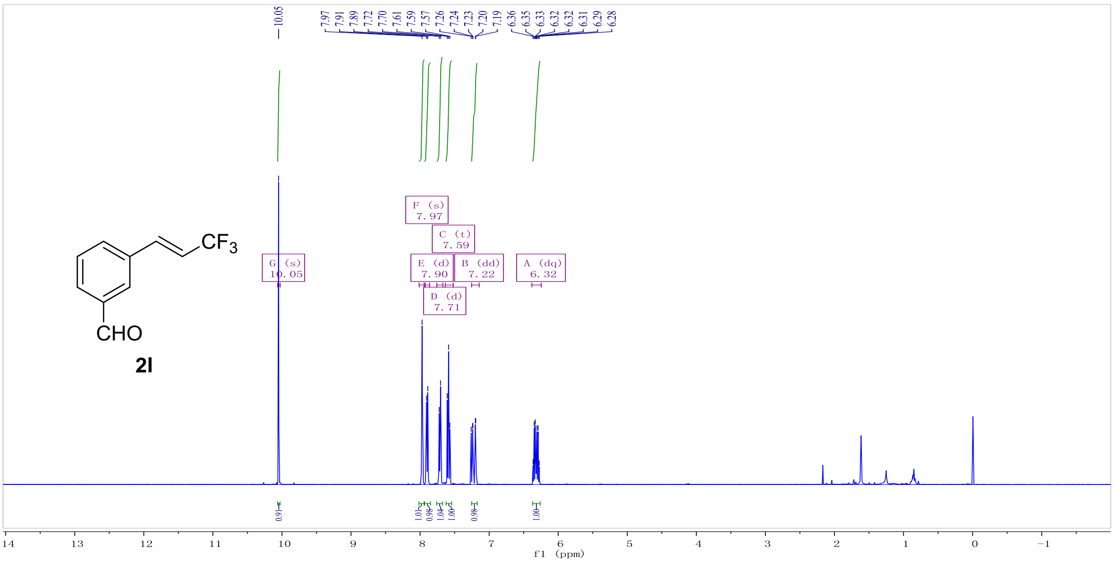
CHO
CF3
2l

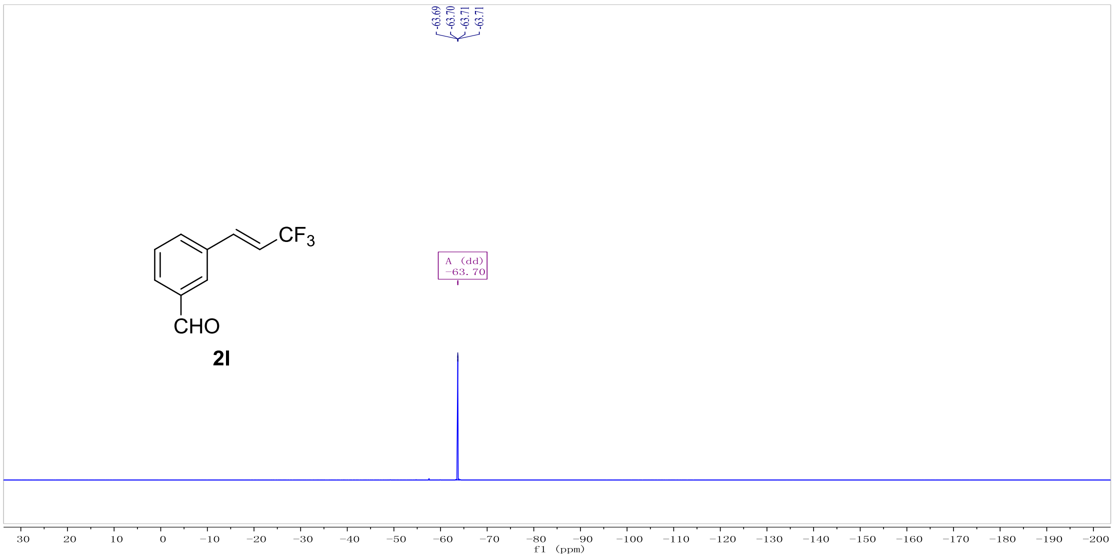
CHO
CF3
2l

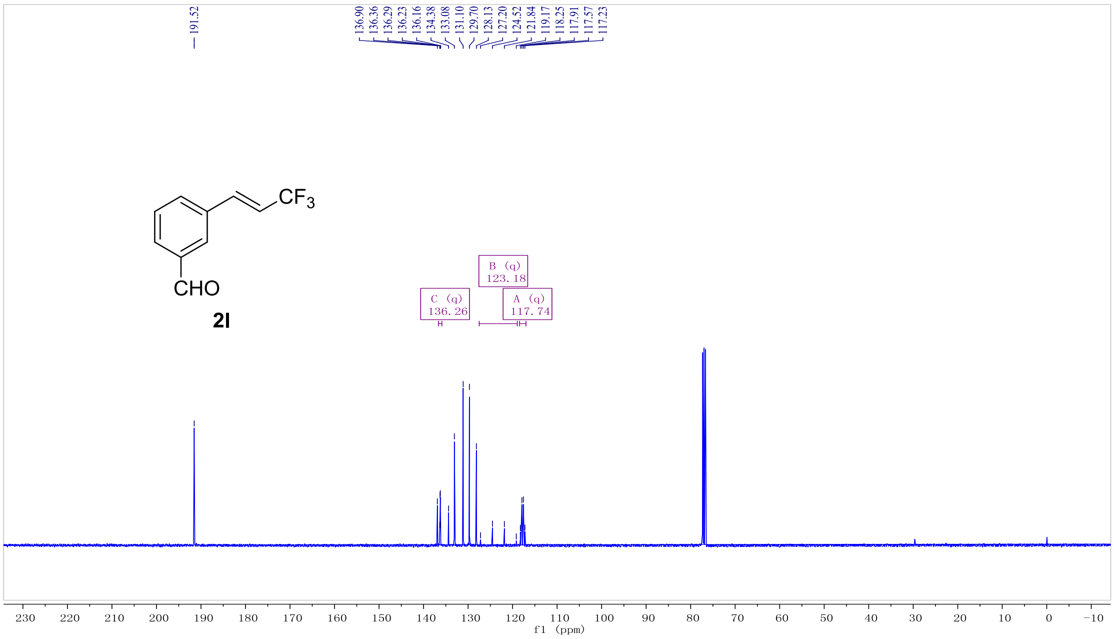
CHO
CF3
2l

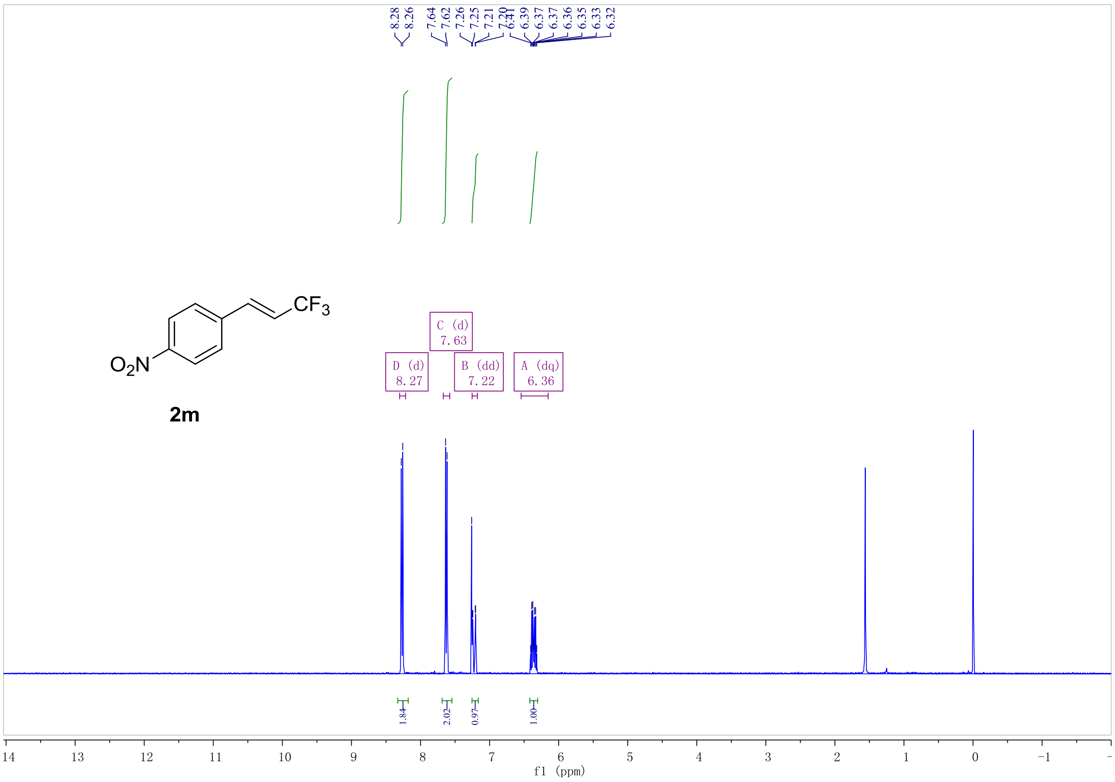
8.28
8.26
7.64
7.62
7.26
7.25
7.21
7.20
6.41
6.39
6.37
6.37
6.36
6.35
6.33
6.32
O2N
CF3
2m
C (d)
7.63
D (d)
8.27
B (dd)
7.22
A (dq)
6.36
1.84
2.02
0.97
1.00
f1 (ppm)

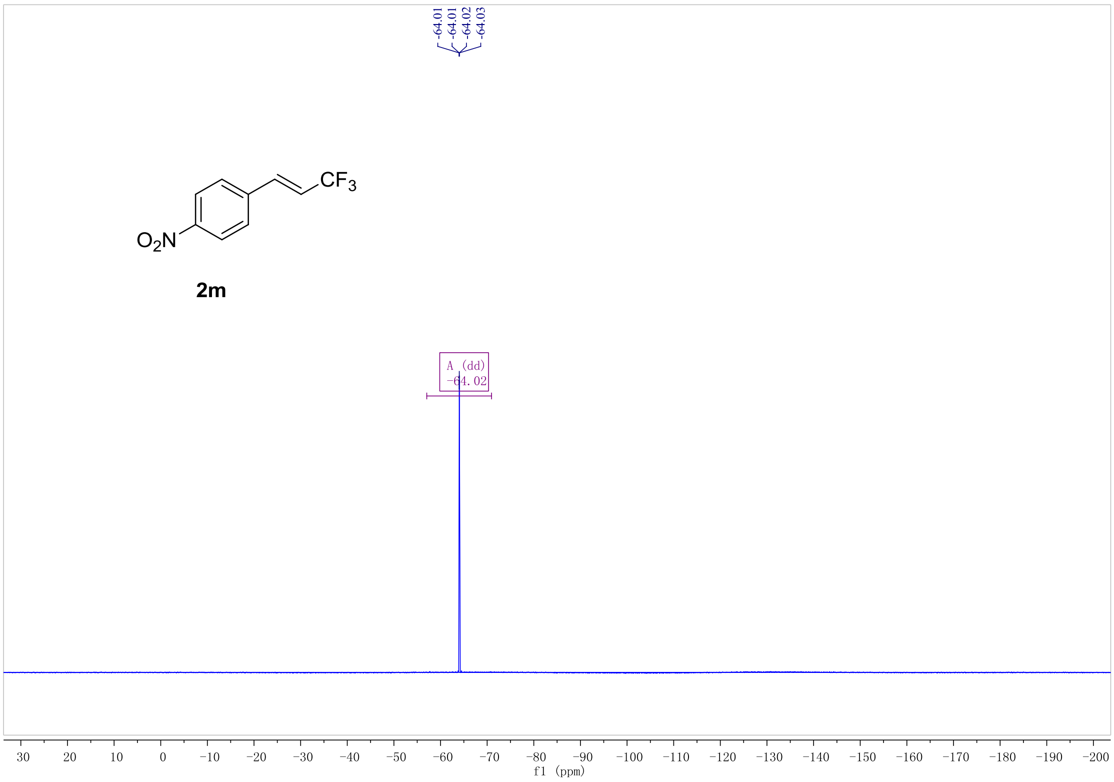
-64.01
-64.01
-64.02
-64.03
O2N
CF3
2m
A (dd)
-64.02
f1 (ppm)

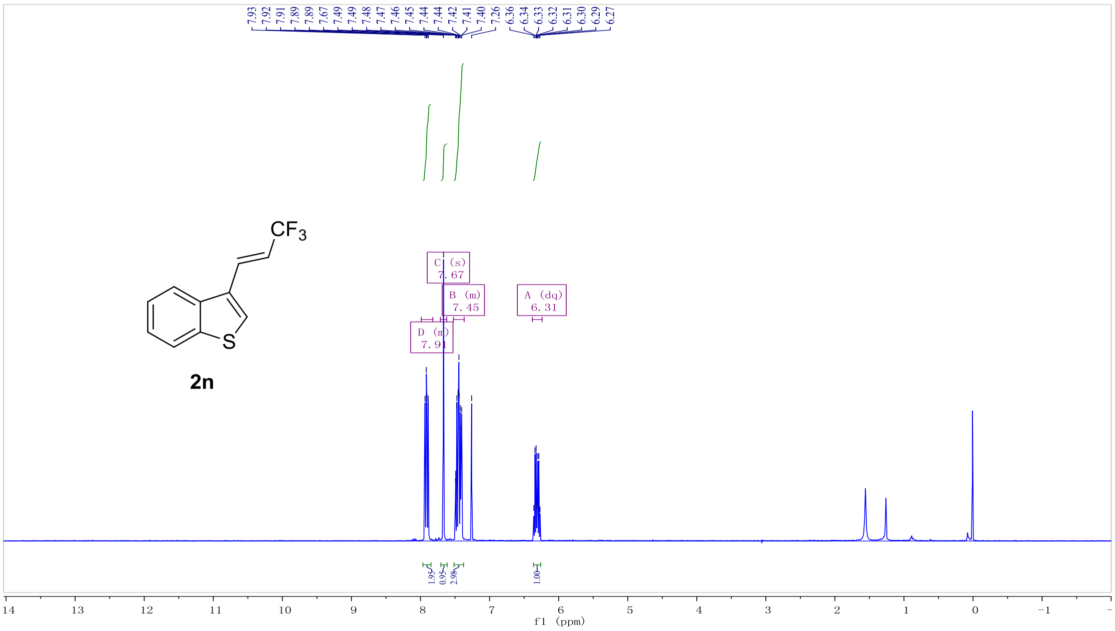
7.93 7.92 7.91 7.89 7.89 7.67 7.49 7.49 7.48 7.47 7.46 7.45 7.44 7.44 7.42 7.41 7.40 7.26 6.36 6.34 6.33 6.32 6.31 6.30 6.29 6.27
C (s) 7.67
B (m) 7.45
A (dq) 6.31
D (m) 7.91
CF_3
S
2n
1.95 0.95 2.98 1.00
f1 (ppm)

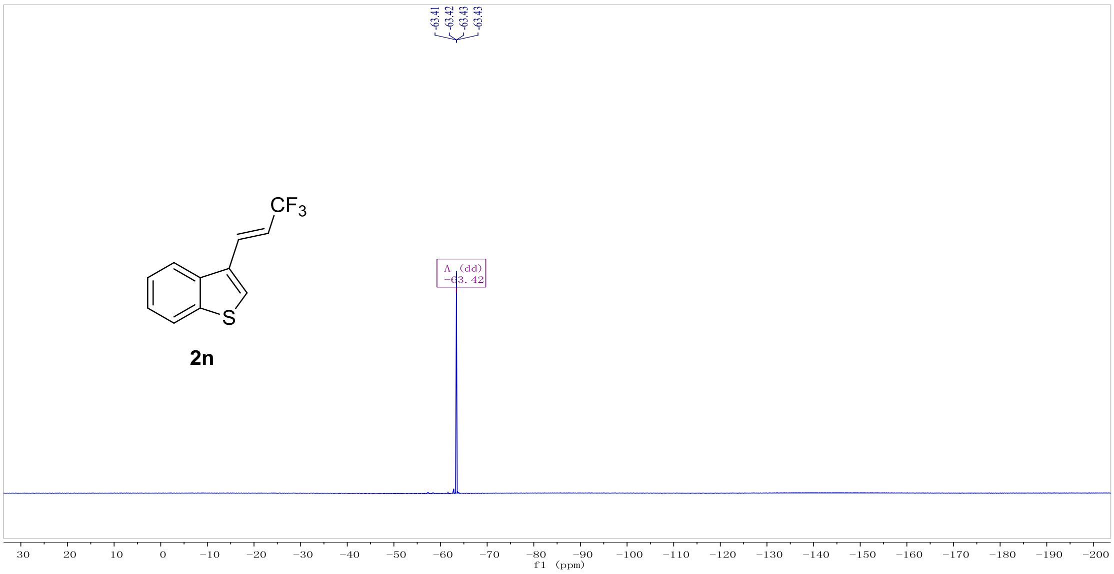
-63.41 -63.42 -63.43 -63.43
A (dd) -63.42
CF_3
S
2n
f1 (ppm)

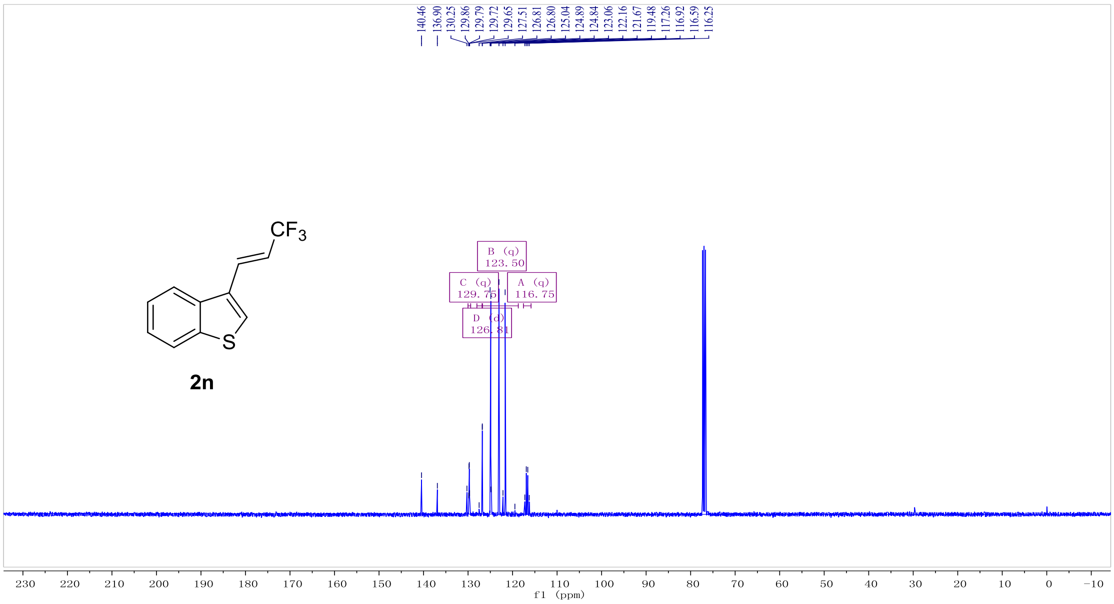
140.46 136.90 130.25 129.86 129.79 129.72 129.65 127.51 126.81 126.80 125.04 124.89 124.84 123.06 122.16 121.67 119.48 117.26 116.92 116.59 116.25
B (q) 123.50
C (q) 129.75
A (q) 116.75
D (d) 126.81
CF_3
S
2n
f1 (ppm)

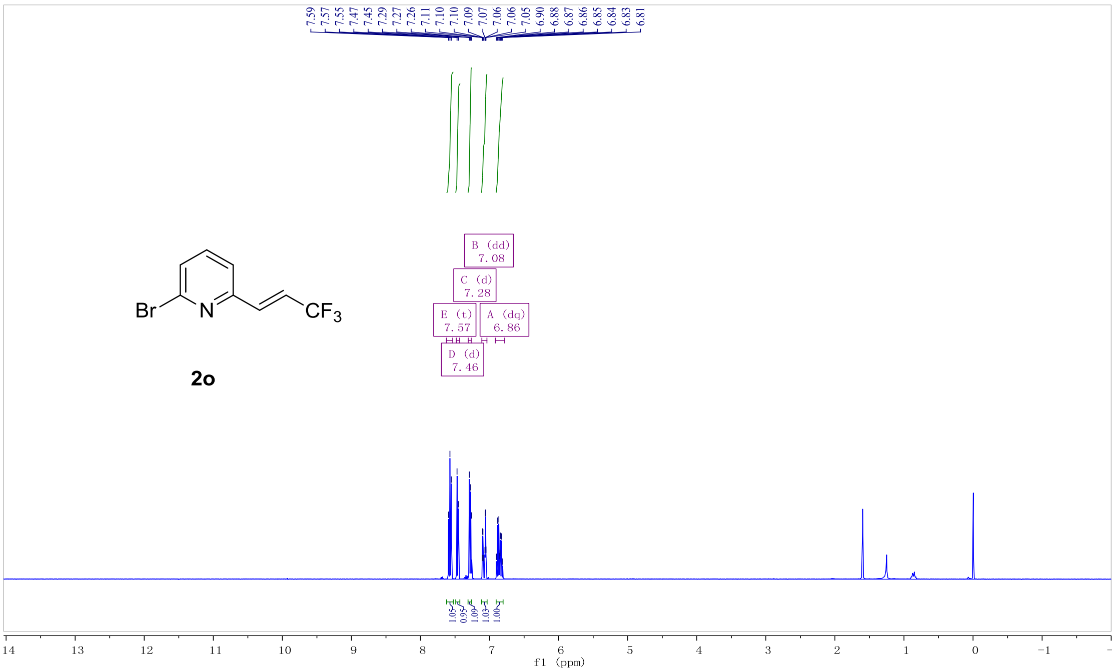
7.59 7.57 7.55 7.47 7.45 7.29 7.27 7.26 7.11 7.10 7.10 7.09 7.07 7.06 7.06 7.05 6.90 6.88 6.87 6.86 6.85 6.84 6.83 6.81
B (dd) 7.08
C (d) 7.28
E (t) 7.57
A (dq) 6.86
D (d) 7.46
Br
N
CF3
2o
1.05 0.95 1.09 1.03 1.00
f1 (ppm)

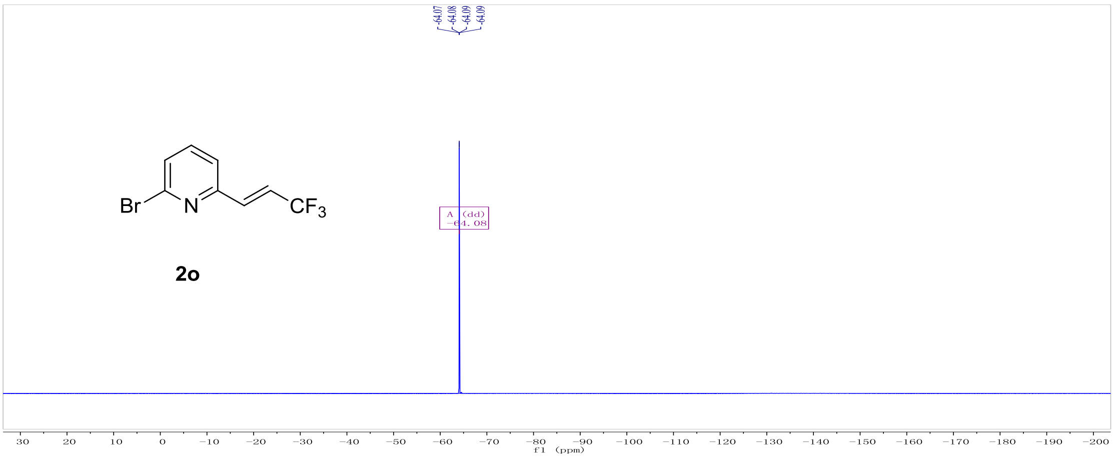
-64.07 -64.08 -64.09 -64.09
A (dd) -64.08
Br
N
CF3
2o
f1 (ppm)

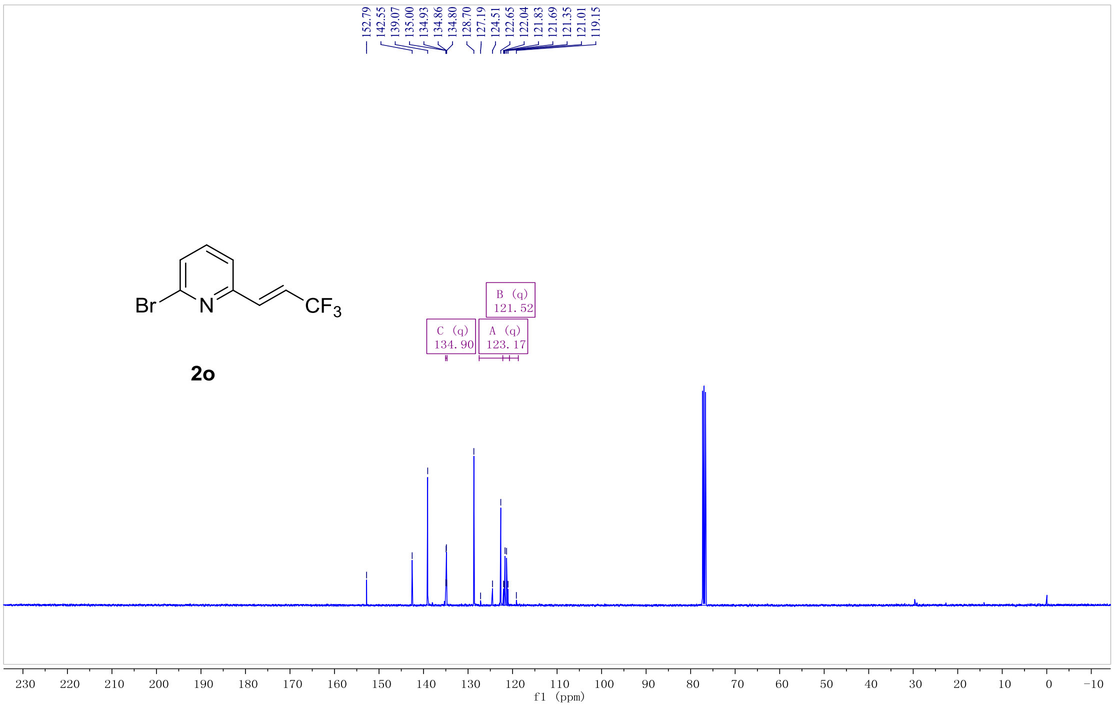
152.79 142.55 139.07 135.00 134.93 134.86 134.80 128.70 127.19 124.51 122.65 122.04 121.83 121.69 121.35 121.01 119.15
B (q) 121.52
C (q) 134.90
A (q) 123.17
Br
N
CF3
2o
f1 (ppm)
